# Probing Membrane Protein Interactions with Their Lipid Raft Environment Using Single-Molecule Tracking and Bayesian Inference Analysis

**DOI:** 10.1371/journal.pone.0053073

**Published:** 2013-01-03

**Authors:** Silvan Türkcan, Maximilian U. Richly, Antigoni Alexandrou, Jean-Baptiste Masson

**Affiliations:** 1 Laboratoire d'Optique et Biosciences, Ecole Polytechnique, CNRS, INSERM U696, Palaiseau, France; 2 Institut Pasteur, Physics of Biological Systems, Paris, France; 3 CNRS, URA 2171, Paris, France; Tohoku University, Japan

## Abstract

The statistical properties of membrane protein random walks reveal information on the interactions between the proteins and their environments. These interactions can be included in an overdamped Langevin equation framework where they are injected in either or both the friction field and the potential field. Using a Bayesian inference scheme, both the friction and potential fields acting on the ε-toxin receptor in its lipid raft have been measured. Two types of events were used to probe these interactions. First, active events, the removal of cholesterol and sphingolipid molecules, were used to measure the time evolution of confining potentials and diffusion fields. Second, passive rare events, de-confinement of the receptors from one raft and transition to an adjacent one, were used to measure hopping energies. Lipid interactions with the ε-toxin receptor are found to be an essential source of confinement. ε-toxin receptor confinement is due to both the friction and potential field induced by cholesterol and sphingolipids. Finally, the statistics of hopping energies reveal sub-structures of potentials in the rafts, characterized by small hopping energies, and the difference of solubilization energy between the inner and outer raft area, characterized by higher hopping energies.

## Introduction

The cell membrane is the interface where the communication between the environment and the cytosol takes place. As such, its structure and time-dependent organization has attracted considerable investigation efforts, both theoretical and experimental, in the form of a large variety of techniques. It is now generally accepted that membranes of living cells harbor areas enriched in cholesterol and sphingolipids, namely lipid rafts, where many proteins important for signaling are concentrated [1]–[13]. These rafts are known to be more densely packed than their surroundings [14] and have been reported to be areas where the membrane thickness is larger [1], [15]. Yet, the inner organization of rafts remains mostly unknown. Rafts have been observed as transient entities of a few tens of nm size which can assemble to yield more stable raft platforms in the range of several hundred nm[4], [6], [10], [16]–[19]. Several hypotheses have been forwarded to explain how the relevant lipids and proteins are recruited in lipid rafts. Some are based on hydrophobic mismatch between the proteins and the surrounding lipids due to the length of the hydrophobic area and/or the hydrophobicity level [1], [13], [20], [21]. However, despite the enormous progress that has been accomplished, novel experimental data on the protein-lipid interactions is still highly desirable.

Using single toxin tracking, we have recently provided new evidence that pore-forming toxins, which need to oligomerize before pore formation, exploit the lipid rafts to concentrate their monomers by recognizing receptors localized in rafts [22]. We have also shown that these pore-forming toxin monomers are valuable probes for visualizing the membrane organization with minimal modifications through the confined motion of their receptors inside lipid rafts, in contrast to other toxins, like cholera toxin, which induce major rearrangements [23].

The ε-toxin of *Clostridium perfingens* (CPεT) is the most virulent toxin of the pore-forming toxin family (lethality of 100 ng/kg in mice) and causes fatal enterotoxemia in livestock [24], [25]. It is secreted by the bacterium in the gut of infected animals in an inactive prototoxin form. A C-terminal and an N-terminal peptide are then cleaved to yield the activated form which is capable of oligomerizing and forming pores in membranes of specific target cells leading to ion leakage and cell death. Structural data have shown that it consists of a receptor binding domain, a domain responsible for oligomerization, rendered accessible after cleavage of the C- and N-terminal peptides, and a domain containing a two-stranded sheet that is thought to be inserted in the membrane to form a β-barrel together with the insertion sheets of six other monomers [26]. Its mechanism of action is the following: recognition of a specific receptor and subsequent oligomerization followed by insertion of a β -barrel forming a pore in the membrane. The CPεT is known to target a 36-kD protein receptor in Madin-Darby canine kidney (MDCK) cells [27], possibly the hepatitis A virus cellular receptor 1 [28].

We have shown that the toxin receptor in MDCK cells is recruited to confinement domains prior to toxin binding, at 37°C, the confinement domain area ranges from 0.01 to 0.8 µm^2^, the diffusivity (averaged over the diffusivity field in each domain and over all the confinement domains) in the domains is 0.13 µm^2^ s^−1^, the motion of the receptor is not purely diffusive but is also influenced by an interaction potential that, if approximated by a spring-like potential, is characterized by a mean spring constant of 0.40 pN.µm^−1^(94 k_B_T.µm^−2^) (averaged over all confinement domains) and that these domains are stable over periods longer than 10 minutes [22]. Most importantly, we have shown that cholesterol oxidase (CHOx) and sphingomyelinase (SMase) treatments drastically decrease the CPεT receptor confinement, whereas the cytoskeleton does not play a direct role in the confinement, which lead us to attribute the confinement domains to lipid raft platforms [22].

Biomolecules evolving in complex and/or time-varying environments reveal information on their physical interactions with their surroundings through the statistical properties of their random walks. Biomolecule motion is often modeled through the unique angle of a pure diffusion process reducing all interactions to a spatially and/or temporally varying friction field. Yet, interactions such as electrostatic interactions, hydrophobic interactions, local binding or specific interactions between biomolecules such as scaffolding biomolecules cannot be fully included in a pure diffusion process or only if the interactions are weak [29]. Indeed, we here demonstrate that the interactions between the biomolecules with the various membrane constituents can be separated into two distinct components: a friction field and an interaction field.

We have demonstrated that Bayesian inference analysis can be used to extract without contact both diffusion and force fields acting on membrane proteins by simply recording the membrane protein trajectories [30], [31]. Confined trajectories are particularly interesting, as the biomolecules explore the same membrane area inside the confinement domain and hence accumulate information on the local physical interactions. However, adaptation of the inference scheme to two further biological processes is necessary to deepen the understanding of the interaction between the receptor and its environment. The first one is linked to slow time-dependent processes. A temporal inference scheme, with strong conditions on its use, has been designed to fill that gap. The second one is linked to local motion transitions where parts of space are under-sampled. We designed an adapted inference scheme to deal with these almost empty spaces.

The purpose of the present work is to combine passive single-molecule motion observations and active cell membrane modifications with new developments of the inference scheme to quantify protein-lipid interactions in raft confinement domains. We employ our new inference approaches to study the ε-toxin receptor dynamics inside lipid rafts. We modify the lipid content of the confinement domain and use a temporally resolved version of the inference to study the evolution of the diffusion and potential fields inside the rafts. Cholesterol and sphingomyelin are shown to play an essential and distinct role in the confinement of the ε-toxin receptor by simultaneously diminishing its average diffusivity and by increasing its confinement. Moreover, we estimate the free energy change of the protein-raft complex due to the removal of cholesterol and sphingomyelin. We furthermore exploit passive events such as hopping, *i.e.* transition from one confinement area to another, to measure the energy needed to overcome the potential barrier between two nearby rafts. We interpret the hopping energy as the solubilization energy difference of the membrane protein between the inner raft and outer raft phase.

## Results

### Bayesian method to extract complex and time-evolving potential structures

Our analysis scheme is based on Bayesian inference performed on individual biomolecule trajectories. We have already shown that Bayesian inference could be used to extract diffusivities and forces from confined trajectories [30], [31]. In these cases, confined trajectories were recorded; diffusivities and forces could be inferred because the proteins kept on moving in the same area allowing efficient information gathering. Here, information is defined as the Fisher Information [32], which is a way (not the only one) to quantify the amount of information that the trajectory carries about the unknown parameters (diffusivity and force fields) upon which the probability of the trajectory depends.

Here, information about the diffusivities and potential fields has to be extracted from trajectories that exhibit inhomogeneous distribution of information due to unvisited or undersampled portions of space of various sizes. These spaces appear, in hopping events, in the area between the rafts and, in the temporal evolution of the confinement, in various parts of the confinement domain during the partial de-confinement. The motion of membrane proteins depends on a variety of factors, including (but not limited to) local electrostatic interactions, local variation of the membrane viscosity or change of lipid content, molecular crowding, dimensionality of accessible space and intermittent specific and unspecific binding to partners. These factors generate a heterogeneous environment whose modeling depends on the characteristic space, time and energetic scales. Here, the temporal scale is on the order of 10 ms, the space scale is on the order of 10 nm and the energetic scale ranges from 0 to 10 k_B_T. At these scales spatial variations of diffusivities can be detected and also, very importantly, the energetic interactions have still sufficient effect to influence the motion of the proteins. Hence, the biomolecule motion can be modeled by overdamped Langevin dynamics:
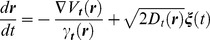
(1)With γ_t_(**r**) the friction field, D_t_(**r**) the diffusivity field, V_t_(**r**) the potential field, and ξ(t) the zero-average Gaussian noise, where the index t stands for possible time dependency. The fluctuation–dissipation theorem gives: D_t_(**r**) = k_B_T/γ_t_(**r**). Note that more complex motion where viscoelasticity is important can be modeled using memory kernels leading to non-Markovian dynamics. In all cases dealt with here, the Langevin equation is a good approximation of the biomolecule motion. This equation is associated to the Fokker-Planck equation:
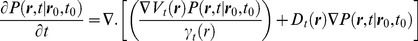
(2)for the two-point conditional transition probability P(**r**,t|**r**_0_,t_0_) for going from the space-time point (**r**_0_,t_0_) to (**r**,t_0_). In both equation (1) and (2), we assumed that the possible temporal variations of both D_t_(**r**) and V_t_(**r**) are slow compared to the dynamics of motion. We show in the following that this assumption is indeed valid.

The area covered by the trajectory is divided into a regular grid of n by n subdomains, in which the gradient of the potential is assumed to be constant. The size of the subdomains is chosen to be equal to two times the average displacement of the protein between frames, to ensure that the motion between frames takes place either inside the same subdomain or produces a move into a neighboring subdomain. The irregular distribution of information, due to the irregular repartition of points, prevents an efficient direct estimation of the forces in the subdomains (the approach we used previously [30]) and imposes the direct inference of the potential [31]. This potential is projected on a function basis and the optimization is performed on the coefficients of the development. Thus, in 2D, the potential reads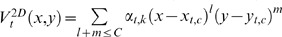 with k = (l+m)(l+m+1)/2+l, (x_t,c_,y_t,c_) the coordinates of the center of mass of the trajectory, and C the order of the polynomial. Polynomial development is not the unique way to develop the potential; here it is sufficient to lead to an efficient inference. Yet, it can be shown that Laguerre polynomials give good results for biomolecule escaping from a confined area and then experiencing free motion. The diffusivity can vary in each subdomain (i,j). Solving Eqn. (2) in subdomain (i,j), with the assumption that 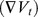 is constant leads to
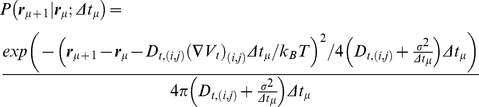
(3)with P(**r**_μ+1_|**r**_μ_,Δt_μ_) the probability of going from **r**_μ_ to **r**_μ+1_ during Δt_μ_, (∇V_t_)_(i,j)_ the gradient of the potential in (i,j), D_t,(i,j)_ the diffusivity in (i,j) and σ the positioning noise, approximated to be the standard deviation of a Gaussian probability of presence of the protein around the detected point. Experimental trajectories contain both static and dynamic positioning noise. Static noise stems from the combine effects of the signal-to-noise ratio in the image and the performance of the emission spot-fitting scheme. Dynamic noise stems from the non-zero acquisition time of the camera that leads to a position averaging effect. Both these effects can be included in a global positioning noise modeled by a Gaussian.

The Bayesian inference features two steps: the derivation of the posterior probability of the parameters P(T|U), *i.e.* the probability that the diffusion and the potential fields have specific values given the observation of the trajectory T, and the sampling of this *posteriori*. Bayes rule states:
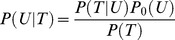
(4)where P(T|U) is the likelihood of the model, *i.e.* the probability of recording a trajectory T when the parameters U take on a specific value, P_0_(U) the prior probability of the parameters, *i.e.* the probability that the parameters U take on a specific value before the experiments is done, which is constant here because there is no prior information on the parameters and P(T) = ∫P(T|U)P_0_(U)dU the evidence of the model, *i.e.* the total probability of the trajectory in the specific model used to perform the inference. As there is no comparison between different models here, P(T) is taken to be constant. The posterior distribution of the parameters reads:
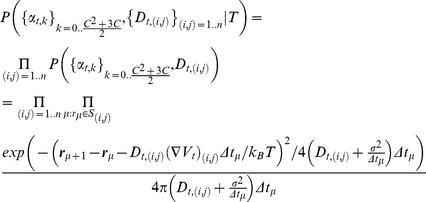
(5)Where (∇V_t_)_(i,j)_ is the gradient of the potential in S_i,j_ (the subdomain(i,j)), Δt_μ_ the time variation between subsequent trajectory points ***r*** *_μ_* and ***r*** *_μ+1_* and where the index μ indicates the times where the protein is inside the subdomain S_i,j_. In order to build this posterior distribution, we used the fact that each subdomain S_i,j_ is independent from the other ones, hence the global posteriori is the product of the local posterior distribution inside each subdomain (first equal sign in Eqn (5)). Furthermore, the motion being Markovian inside each subdomain S_i,j_, the local posterior distribution is the product of the probability of going from one point in the subdomain to another one (second equal sign in Eqn. (5)). Finally, the second step of the inference (the sampling) consisted in using the Maximum *A Posteriori* (MAP) estimator [31], *i.e.* the point in parameter space maximizing the global posterior distribution, to extract the diffusivity and potential fields in the rafts. In the numerical implementation of the inference, the positions are expressed in µm, the diffusivity in µm^2^·s^−1^, and the potential in k_B_T. Further explanation on the choice of the estimator is given in Document S1 (Section C2, Fig S7).

### Temporal variations of diffusivity and potential fields

In various biological systems the diffusion, force and potential fields can vary with time. Equation 1 cannot, in all cases, be associated to the Fokker-Planck equation (Eqn. 2) because fast temporal variations of the diffusion or the potential would drive the Langevin equation out of equilibrium, and the scheme would no longer apply. Furthermore, even if the previous association were possible, it is not necessarily sufficient to allow the inference, for example an insufficient sampling of space by the trajectory would prevent efficient mapping of the diffusivity and potential fields. Three time scales can be associated to the motion and the inference:

- τ_m_ = L^2^/D the characteristic time of the confined motion, *i.e.* the typical time required to move a significant distance inside the confinement area. Here, L is the typical size of the confining domain and D the average diffusivity in the domain. A typical order of magnitude in our case is τ_m_≈1 s.
- τ_inf_ = τ∼{<N_i,j_>≥Ñ} the characteristic time needed to have an average number of points inside each mesh square superior to Ñ, the number of points required to obtain meaningful inferred values. In the previous applications of the inference [22], [31], Ñ≥15 lead to efficient estimation of the parameters.
- τ_V,D_ the characteristic time of the potential and diffusion field variations, *i.e.* the time needed for a significant local (in space) variation of the one of the two fields.

A necessary condition for the use of the inference is
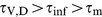
(6)which means that the time window on which the inference is performed is large compared to the time it takes the protein to move from one side of the confinement domain to the other and that during that time the values of the diffusion and potential field have not changed significantly. To ensure that the fluctuation-dissipation relation remained true inside this time window, the duration of the window was chosen to ensure that its doubling would not lead to relative changes of more than 20% of the inferred values. Experimentally, the variation of the diffusivity and potential fields were sufficiently slow so that the classical Fokker-Planck association could be performed. The inference was applied on a sliding temporal window of duration τ_inf_ = 40 s with a shift of 5 s between each measure. The sliding window duration lead to a N_i,j_ varying from 15 to 100 with variations during the evolution of the inference and variations between rafts. Depending on the cells, some slow drifts have been observed. This was corrected by sliding a temporal window of duration τ_inf_ after subtracting the drift motion from the average position of the biomolecules. Note that the correction of the drift can also be achieved inside the inference scheme by adding a drift velocity in the inference model that can be inferred and removed from the trajectory.

**Figure 1:**
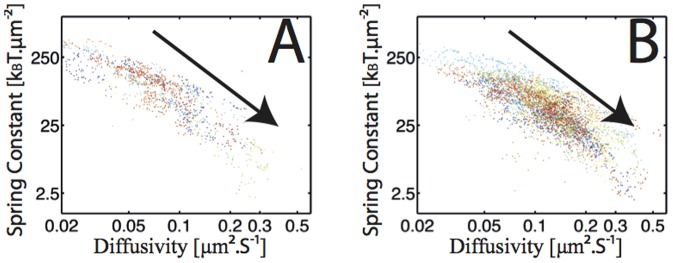
Evolution of the average diffusivity and spring constant of the receptor with the addition of cholesterol oxidase and sphingomyelinase. In (A) the evolution with cholesterol oxidase and in (B) the evolution with sphingomyelinase. The evolutions are plotted in the spring constant versus diffusivity plane. The plot is shown in log-log scale for display purposes. Each experimental evolution is associated to a specific color. The black arrow indicates the temporal evolution for all experiments.

We used ε-toxins of the bacterium *Clostridium perfringens*, labeled with rare-earth doped, non-blinking 30–50 nm Y_0.6_Eu_0.4_VO_4_ nanoparticles [33], [34], [35]. The ε-toxins bind to specific receptors in MDCK cells [28]. The nanoparticle emission thus allows tracking the ε-toxin receptors with a wide-field microscopy setup [22]. CHOx and SMase are commonly used to oxidize cholesterol to cholestenone and transform sphingomyelin to ceramide, respectively, thereby destabilizing lipid rafts [1], [36], [37], [38]. To understand the interactions between the ε-toxin receptor and the surrounding lipids, we studied the temporal evolution of the diffusion and potential fields in the confining raft domain with the addition of CHOx or SMase by single-molecule tracking of the *same* receptor during incubation over several minutes (Fig. 1, Document S1 D2, Fig S19, for harmonic bias correction). In both experiments, the average diffusivity rises with incubation time. There are strong fluctuations during this rise (Fig. S1, S2). The average diffusivity changes from D_i_ = 0.063±0.01 µm^2^·s^−1^ (N_CHOx_ = 27) to D_e_ = 0.18±0.02 µm^2^·s^−1^ (N_CHOx_ = 27) after adding CHOx and from D_i_ = 0.066±0.006 µm^2^·s^−1^ (N_SMase_ = 40) to D_e_ = 0.27±0.02 µm^2^·s^−1^ (N_SMase_ = 40) after adding SMase.

To verify that the changes in diffusivity are indeed due to the destabilization of the lipid raft environment and not to a secondary effect on the whole cell membrane, we performed control experiments on the transferrin receptor which is a well-known non-raft protein [39]. We labeled transferrin molecules with Y_0.6_Eu_0.4_VO_4_ nanoparticles, incubated them with MDCK cells and, after binding of the labeled transferrin molecules to their receptor, recorded transferrin receptor trajectories before and after addition of CHOx. The receptors show hop diffusion [39] with a mean diffusivity of 0.15±0.02 (N = 26) µm^2^/s before and 0.12±0.02 (N = 16) µm^2^/s after incubation with CHOx. We furthermore applied a Kolmogorov-Smirnov test that showed that there is no significant difference between the two diffusivity distributions.

The potential also strongly changes during the incubation time with the enzymes (Video S1, S2, S3 and Fig. S1). Both CHOx and SMase lead to a decrease in receptor confinement. If the potentials are approximated by harmonic ones, as in Ref. [22], the average spring constant after adding CHOx changes from k_i_ = 237±44 k_B_T·µm^−2^(N_CHOx_ = 27) to k_e_ = 35.4±7.7 k_B_T·µm^−2^(N_CHOx_ = 27) and, after adding SMase, from k_i_ = 206±90 k_B_T·µm^−2^ (N_SMase_ = 40) to k_e_ = 10.5±4.1 k_B_T·µm^−2^(N_SMase_ = 40). The simultaneous evolution of the average diffusion and confining potential after the addition of CHOx and SMase is shown in Figs. 1A and 1B, respectively. In both cases, the biomolecule evolves in the diffusivity-spring constant (k-D) plane starting in the high k-low D region and finishing in the low k-high D region. All evolutions follow a similar behavior with strong variations. This demonstrates that cholesterol and sphingomyelin contribute to both the friction and the interaction field in which the receptor evolves.

If we assume that the potential values inferred before and long after (so that the potential no longer varies with time) the CHOx or SMase treatment are close to statistical equilibrium, we can evaluate the free energy difference of the receptor-lipid system (Materials and methods). We find ΔF_cho_ = −1.9±0.2 k_B_T (N_Coase_ = 27) and ΔF_sphi_ = −2.4±0.14 k_B_T (N_SMase_ = 40) after CHOx and SMase addition, respectively. However, a quantitative comparison is not easy for several reasons: i) we have measured the total amount of cholesterol left after its oxidation to cholestenone and hydrogen peroxide, and the total amount of sphingomyelin left after breaking it down into phosphocholine and ceramide (Document S1) not the amount left in membrane rafts. ii) These reactions that are facilitated by CHOx and SMase are not just responsible for the removal of cholesterol and sphingomyelin, but additionally contribute to raft destabilization via the production of cholestenone and ceramide, respectively [40], [41].

### Hopping energies extracted from experimental trajectories

The definition of the hopping energy is not without ambiguities and may vary depending on the biological system. Here, we define the hopping energy between two confinement areas as the difference between the maximum value of the potential energy along a line linking the two minima of potentials in the two wells and the global minimum value of the potential within the two wells (Document S1, section B, Fig S3, S4, S5, S6). Note that, depending on the geometry of the trajectories, simple numerical schemes have to be designed to search for the minima of the potentials in the different wells. In the case of hopping between a unique well and free motion, the hopping energy is defined as follows: for 1D trajectories, it is the highest energy difference between the limits of the confining domain and the minimal potential energy in the well. For 2D trajectories, we define the hopping energy as the difference between the average value of the potential at the limits of the confining domain and the minimal value of the potential energy in the well.

The scheme performance was evaluated on numerical trajectories matching both experimental conditions and relevant conditions for other biological systems (Document S1, section C, Fig S7, S8, S9, S10, S11, S12, S13, S14, S15, S16, S17, S18). We used a fourth-order polynomial potential (up to sixth order for large-distance hopping) both for the simulated trajectories and for the inference, which is sufficient to describe a potential with two minima. Simulations were used to quantify the behavior of the inference, *i.e.* the convergence, the bias and the error on the estimated parameters and to directly assess the error in the determination of the experimental hopping energy. The Fisher information [32], [42] is not accessible due to the vast parameter space. Hence, the error on the estimated parameters can only be accessed by simulations matching experimental conditions.

**Figure 2:**
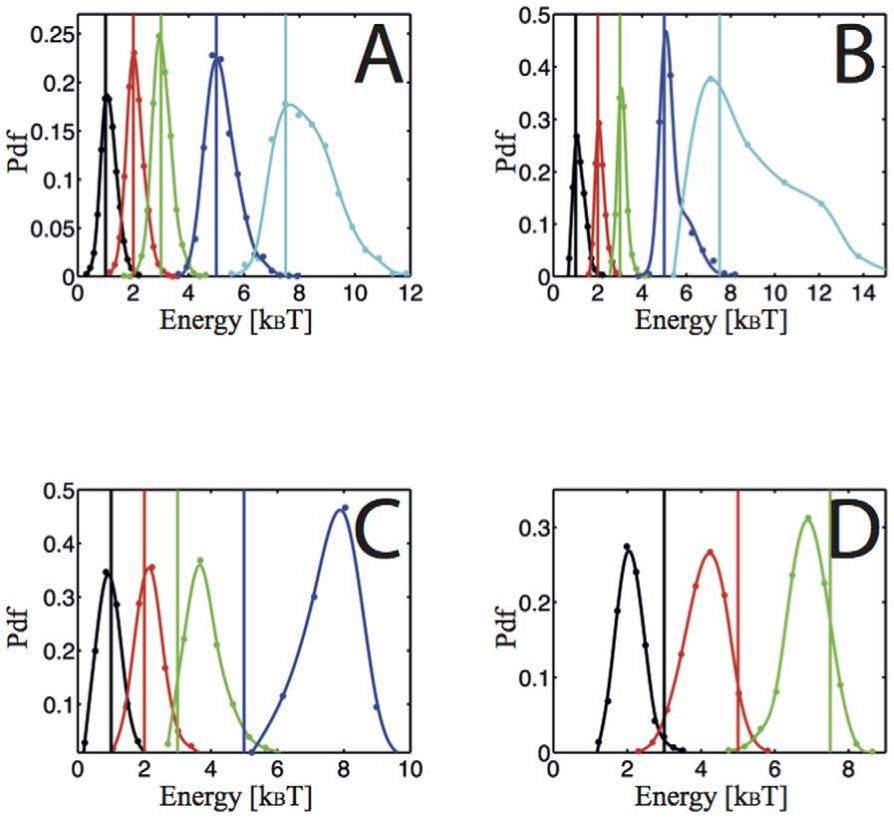
Evolution of the MAP Statistics with the hopping energy. The vertical line is the input value used in the trajectory generation; the corresponding statistics share the same color. 2000-point trajectories; acquisition time: 25 ms. A) 1D trajectories, hopping between 2 wells separated by 400 nm with 0.025 µm^2^·s^−1^ diffusivity. B) 2D trajectories, hopping between 2 wells separated by 300 nm with 0.025 µm^2^·s^−1^ diffusivity and harmonic lateral confinement with spring constant 200 k_B_T·µm^−2^. C) 1D trajectories, hopping between a unique harmonic well and free motion. Confinement radius: 100 nm; diffusivity: 0.035 µm^2^·s^−1^. D) 2D trajectories, hopping between a unique harmonic well and free motion with a confinement radius of 100 nm; diffusivity: 0.025 µm^2^·s^−1^.

For different values of the hopping energies, for 1D and 2D trajectories, we generated a large number of numerical trajectories and then plotted the statistics (probability density function, Pdf) of the hopping energies extracted with our inference scheme (Fig. 2A,B). The inference scheme is able to catch the hopping energy between two confining wells in both 1D and 2D, it is unbiased and the width of the distributions has low values for small and average hopping energies. Interestingly, even if the hopping region is under-visited by the biomolecule, there is enough information in the two confining areas to catch the hopping energy (Document S1, section C.5, Fig S11, S12). Inferences performed on simulated trajectories of escaping biomolecules from a unique well exhibit a more complex behavior (Fig. 2 C, D). As the hopping energy increases, the inference becomes biased for 1D trajectories. For 2D trajectories, a bias appears for low hopping energies. Yet for both cases, the effect is deterministic and can be analytically compensated.

**Figure 3:**
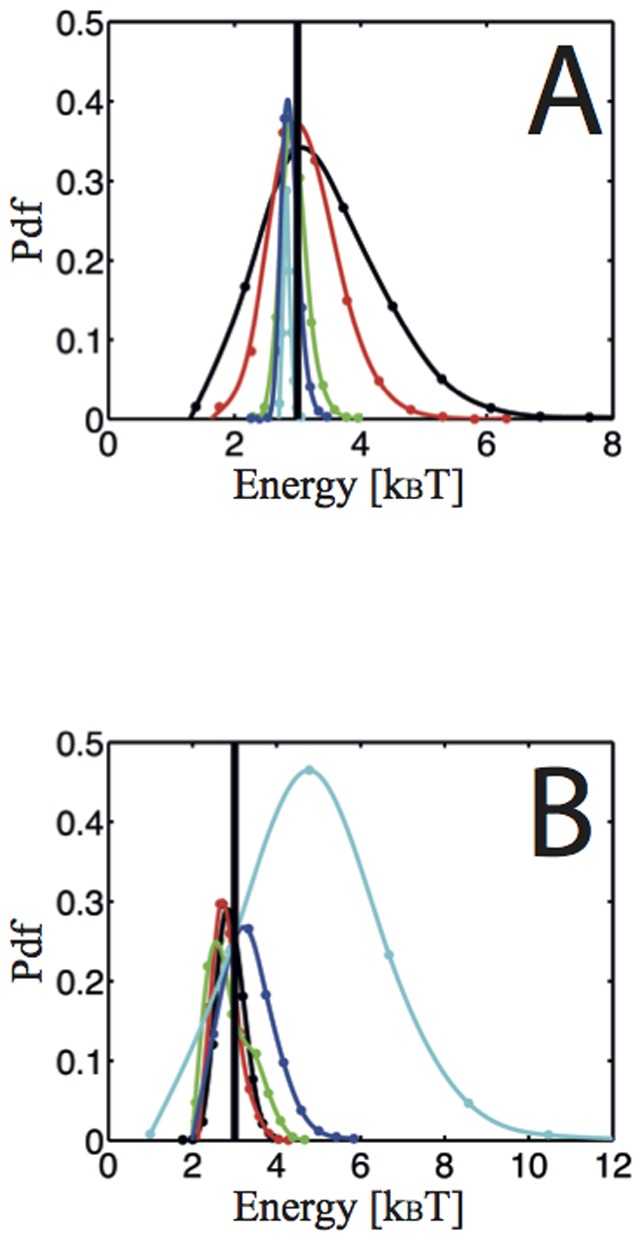
Evolution of the MAP statistics with trajectory length and positioning noise. In (A) the evolution with length in (B) the evolution with noise. Input hopping energy: 3 k_B_T (shown by the thick vertical black line); distance between the two wells: 200 nm; diffusivity: 0.025 µm^2^·s^−1^; acquisition time: 25 ms. A) Trajectory points: 500 (black), 1000 (red), 5000 (green), 10000 (blue), 50000 (cyan). B) The apparent diffusion coefficient due to positioning noise D_noise_ = σ^2^/Δt, with σ the standard deviation of the positioning noise, takes on different values with respect to the diffusion coefficient D: α = D_noise_/D with α = 1.4% (black), α = 5.8% (red), α = 16% (green), α = 64% (blue), α = 144% (cyan). 2000-point trajectories.

The length of the trajectories and the positioning noise are two important parameters acting on the scheme efficiency. Examples of the evolution of the inference performance with these two parameters are shown in Fig. 3. The inference results remain unbiased even for relatively short trajectories, however the width of the distribution increases. The minimum number of points required to obtain a good inference depends on the hopping energy and on the diffusivity field values. Interestingly, the inference is able to determine the hopping energy even with high positioning noise (Fig. 3B). This offers the possibility of greatly shortening acquisition time to study fast dynamics and local low energy hopping.

**Figure 4:**
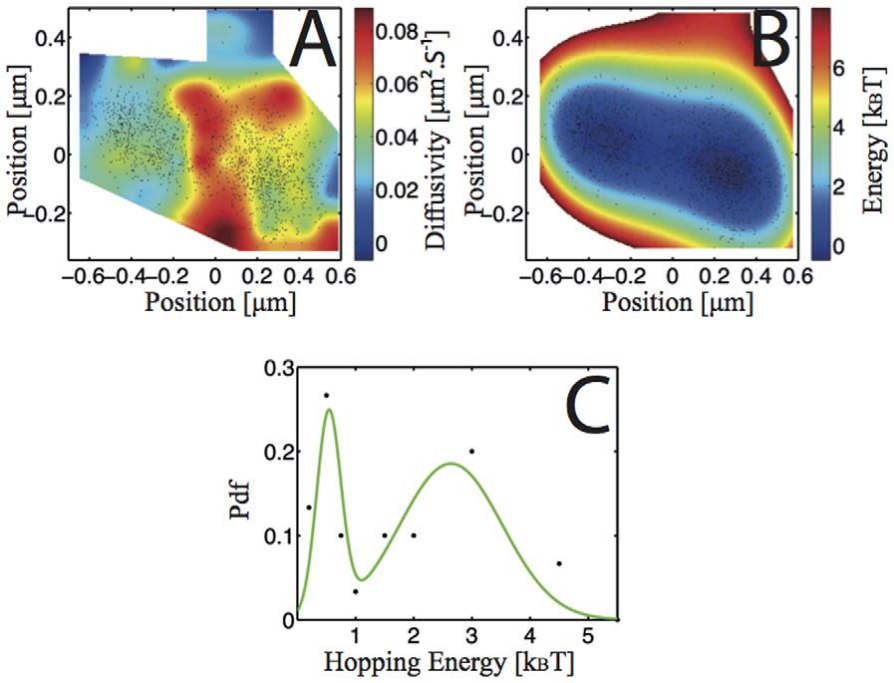
Hopping events with trajectory points as black dots. The duration of the trajectory is 1321 frames, *i. e.* 67.8 s. A) Diffusivity map of the membrane area where the receptor moves. The diffusivity field was generated by a bi-harmonic interpolation of the inferred diffusivity values on the mesh. B) Interaction energy map acting on the receptor. C) Statistics of hopping energy. Black points are experimental results and the green line is a smoothing spline intended as visual aid.

In Figs. 4A–B, we show the trajectory points of a receptor undergoing hopping. The ε-toxin receptor goes from a confining area of approximate diameter 200 nm to another of approximate diameter 400 nm (Figs. 4A–B). Note that the dissociation constant of the ε-toxin from its receptor is very low K_d_ = k_off_/k_on_ = 3.8±1.9 nM [43]. In fact, we never observe dissociation and departure of a labeled toxin after binding to its receptor. This is true even for experiments performed under application of an external force except for rare occasions under high force values of about 4.0 pN [44]. It is therefore extremely unlikely that the jumping events we observe are due to toxin dissociation and subsequent binding to an adjacent receptor rather than receptor hopping from one raft domain to an adjacent one, especially considering that, in the large majority of cases, we observe multiple back and forth hopping events (see Figs. 4A, S20, S21, S22).

As in every hopping event, the hopping area is under-visited compared to the confining area. The inferred diffusivity and potential field are displayed in Fig. 4 A and B, respectively. The potential field exhibits a double-well structure, with two well-defined minima and a local maximum area separating the two wells. Interestingly, there is a detectable rise of diffusivity in the area between the two confining wells. Note that the only previous observations of hopping events concern jumping over cytoskeleton barriers [45]. The hopping events between rafts are rare but reproducible events. We recorded over 600 trajectories corresponding to a full recording time of over 20,000 s leading to an effective approximate ratio of 5% of analyzable trajectories showing hopping. The average duration of the analyzed trajectories was 2400±225 frames corresponding to 123±12 s with an average static positioning noise of 30 nm, hence well in the zone where the inference is very efficient and approximately unbiased. For the inference, we took σ = 30 nm and verified that using σ = 45 nm induces a change in the inferred values of less than 20%.

The distribution of the hopping energies extracted from experimental hopping trajectories (Document S1, section D.3, Fig S20, S21, S22) is shown in Fig. 4C. The experimental results show two kinds of hopping events: (i) multiple receptor hops from one potential well to the adjacent one related to a low average hopping energy <ΔE_h_> = 0.54±0.05 k_B_T (N = 18). These events seem to be related to multiple structures within the lipid raft. (ii) One or few hopping events shifting the trajectory from one potential well to another one (Fig. 4A–B and Fig S20, S21). In this case, the average hopping energy is higher, <ΔE_h_> = 2.64±0.25 k_B_T (N = 15). These hopping events can be associated to the receptor jumping from one raft to another.

## Discussion

The novelty of our approach lies in the simplified model of the interactions between membrane proteins and their environments. In this model, the ε-toxin receptor motion in its raft environment is that of a random walker in a field of friction γ_t_(**r**), leading to a diffusion field D(**r**), and submitted to an interaction field V(**r**) which includes the local electrostatic interactions, hydrophobic interactions, possible lipid-protein specific or non-specific interactions, local tension and curvature effects. The overdamped Langevin equation can model this biomolecule motion and the associated inference scheme yields a quantitative measure of the interactions between the biomolecule and its environment. Thus, the inference scheme extracts and differentiates the two contributions, D(**r**) and V(**r**).

The temporal inference scheme highlights the combined effect of lipid modifications on both the diffusion and potential fields. Adding CHOx or SMase leads to a simultaneous change of the diffusion and the potential. The broad distribution of the receptor confinement domain sizes and the large variations of potentials and diffusion fields after incubation with CHOx or SMase prevent from fully exploiting the temporal evolutions of these quantities. On subsets of data, however, we observed larger variations of diffusivities in the center of the rafts than at the borders and a higher harmonicity of the potential, after the addition of SMase, whereas the addition of CHOx leads to a spatially more homogenous evolution. This suggests a non-homogeneous distribution of the different kinds of lipids inside the raft as hypothesized in Ref. [22]. However, additional experimental data are needed to investigate this aspect of lipid organization.

We have shown in our previous work with latrunculin B and nocodazole control experiments that actin filaments and microtubules do not play a role in the confinement [22]. The structure and evolution of the inferred potentials further confirms the absence of cytoskeleton involvement. Indeed, we expect confinement due to the cytoskeleton to manifest itself in a fast rise of the potential values near the edge of the confinement areas, which is not what we observe. The ε-toxin receptor confinement is thus mostly due to the composition and spatial organization of the lipids surrounding the protein inside the raft structure. We observed no events of receptors leaving the raft-phase into the non-raft phase. Hopping behavior thus seems possible only if another raft with similar lipid organization is present in the neighborhood. Therefore, the rarity of hopping events can be explained by the fact that most of the time there is no adjacent raft to jump to.

Figure 4C shows two types of hopping events corresponding to different magnitudes of the hopping energy and to different associated geometric characteristics (Document S1, section D.3). Large hopping energy values are relevant to evaluate the energy difference between the center of the raft and its outer parts. In this case, the hopping energy can be interpreted as
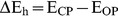
(7)with E_CP_ (CP: Center Potential) and E_OP_ (OP: Outer Potential) the interaction energy between the protein and the lipid organization at the center and border of the potential well, respectively. This hopping energy can thus be interpreted as the solubilization energy difference (not the free energy) of the protein between the lipid organization at the center and the border of the confining well.

Interestingly, the hopping energies are not very high (<ΔE_h_> = 2.64±0.96 k_B_T) hence reinforcing the idea that similar lipid contents must be present near the main confining well to lower the energy sufficiently to allow a hopping event. This is confirmed by the fact that, in trajectories remaining confined in the same raft, the receptor often moves in regions where the interaction energy rises above 6 k_B_T. This leads us to think that the solubilization energy difference between the raft and the non-raft phase must be at least 6 k_B_T. This value is compatible with a report indicating that approximately 10 k_B_T are required to insert a membrane protein into a 5-nm thick lipid membrane [46]. Indeed, we expect that the energy required to insert a protein inside the membrane should be higher than that required to displace it from the raft to the non-raft phase.

Small hopping energies reveal sub-structures in the raft. They are usually found in larger rafts than for the case of confined motion without hopping. Large raft structures are believed to be generated by coalescence of multiple small rafts and by reorganizing the lipid structure within the newly formed raft [1]. The double-well structures we observe may thus reflect the coalescence of two rafts. We also observed intermediate potential structures with a highly asymmetric main potential well but without a second minimum (Document S1, section D.3.d, Fig S23) that seem to confirm the existence of merging behavior. These data were not included in the hopping energy measurements.

Finally, we propose possible extensions of the scheme and further experiments to investigate the raft structure. A non-homogenous lipid distribution inside the rafts may be detected from the inferred diffusion field of the protein using an appropriate mesh definition and a Bayesian criterion to decide what is the most probable organization. Experimentally, tracking of individually tagged lipids should improve our understanding of the lipid organization in the raft. Furthermore, long-term recording (up to 1 hour) of protein trajectories appears necessary to quantify the evolution of diffusion and potential fields as the raft structure evolves. Local unspecific tagging of the cell membrane would be useful to measure the motion of the membrane and compensate for it.

## Materials and Methods

### Coupling of toxins to nanoparticles

We coupled APTES-coated Y_0.6_Eu_0.4_VO_4_ nanoparticles to ε-prototoxins (CPεpT), the non-cleaved precursors of CPεT, or to CPεT, via the amine-reactive cross-linker (bissulfosuccinimidyl) suberate (BS_3_, Pierce Protein Research), as reported in Ref. [22]. The advantage of the CPεpT is that it couples to the same receptor as CPεT, but cannot form oligomers. The NP-protein coupling ratio can be adjusted by varying the ratio of the toxin concentration to the nanoparticle concentration. A BCA test used to determine the amount of toxin after the coupling process, showed a toxin∶nanoparticle coupling ratio of 3∶1. Nanoparticles without toxins do not bind to the cells and are rinsed away. Given the size of the NPs and the presence on non-functional toxins after conjugation [22], it is improbable that more than one functional toxin is present on the same area of the NP surface allowing simultaneous binding to more than one receptor. We therefore estimate that the fraction of NPs bound to more than one receptor is negligible.

To label transferrin receptors, we first prepared streptavidin-coated nanoparticles, as described above, using APTES-coated Y_0.6_Eu_0.4_VO_4_ nanoparticles and the cross-linker BS3. A BCA test determined a streptavidin∶NP coupling ratio of 11∶1. We then incubated a 400 µL NP-streptavidin solution with a concentration of 0.1 mM in vanadate ions with 100 µL of a 1 mg/mL transferrin-biotin (Invitrogen) solution at 37°C for 30 minutes to obtain transferrin labeled with Y_0.6_Eu_0.4_VO_4_ nanoparticles.

### Single Molecule Tracking

The experimental conditions were the same as in Ref. [22]. Tracking experiments were performed with a wide-field inverted microscope (Zeiss Axiovert 100) equipped with a 63×, NA = 1.4 oil immersion objective and a EM-CCD (Roper Scientific QuantEM:512SC). NPs were excited with an Ar^+^-ion laser using the 465.8 nm line. Emission was collected through a 617/8M filter (Chroma). Confluent cells on coverslips were incubated with 0.04 nM NP-labeled *Clostridium perfingens* ε-prototoxin (CPεpT) for 10 minutes at 37°C. The concentration is low to avoid oligomerization and observe single NPs (<10 per cell). The sample was then rinsed three times to remove non-bound toxins and nanoparticles. We recorded images at a frame rate of about 20 Hz (exposure time: 50 ms; readout time: 1.3 ms) and an excitation intensity of 0.25 kW/cm^2^ at 37°C. The toxin receptor position in each frame was determined from a Gaussian fit to the diffraction pattern of the nanoparticles with a home-made Matlab V8.2 (Mathworks, Natrick MA) algorithm.

The average duration of the ε-toxin receptor trajectories is 4300±740 frames corresponding to 221±38 s for the cholesterol oxidase experiments and 9500±850 frames corresponding to 487±44 s for the sphingomyelinase experiments. The average duration of the hopping experiments is 2400±225 frames corresponding to 123±12 s. The mean total photon number per nanoparticle label in each frame is 70 photons, the average signal-to-noise ratio is equal to 10, and the average static positioning noise is 30 nm corresponding to an apparent diffusivity due to noise of D_noise_ = 0.018 µm^2^·s^−1^. A discussion on the determination of the static positioning noise can be found in the supporting material of Ref. [22].

We demonstrated in Ref. [22] that binding of the nanoparticle-labeled ε-prototoxins on MDCK cells is specific. We here verified that binding of the nanoparticle-labeled transferrin is also specific: We imaged 25 different cell areas that showed binding of 60 transferrin-NP conjugates versus binding of only 8 streptavidin-NP conjugates without transferrin in the same conditions.

### Cell Culture

Madin-Darby canine kidney (MDCK) cells were cultured in (DMEM, 10% fetal calf serum (FCS), 1% penicillin-streptomycin) culture medium (CM) at 37°C. For tracking experiments, cells were trypsinated two days before and transferred onto acid-bath treated glass microscope coverslips and grown until confluent. The medium was replaced by an observation medium (OM) (HBSS+10 mM HEPES buffer, 1% FCS) just before the tracking experiment, which lasted less than 1 h. Pharmacological treatment of the cells were performed in a minimal medium (MM) (HBSS+10 mM HEPES buffer).

### Pharmacological Treatments of the Cell

Where mentioned, we incubated cells with either 20 U/mL cholesterol oxidase (Calbiochem) or 10 U/mL sphingomyelinase (Calbiochem) in HBSS+10 mM HEPES for 30 minutes. A cholesterol quantification kit (Invitrogen) was used to determine successful cholesterol digestion on lyzed cells before and after incubation.

To determine the amount of sphingomyelin broken down by sphingomyelinase, a sphingomyelinase quantification assay kit (Amplex® Red, Invitrogen) was used. We found 30% less cholesterol and 45% less sphingomyelin in the cell lysates that had been incubated with cholesterol oxidase and sphingomyelinase, respectively. Note that all experiments with cholesterol oxidase and sphingomyelinase incubation were performed on the same day on cells grown in the very same conditions, so that the initial receptor motion characteristics (diffusivity, spring constant and domain area) would be as close as possible for the two types of experiment.

### Free Energy measure

Assuming that the inferred values of the potential can approximate the statistical equilibrium states before and long after the addition of CHOx or SMase, we can evaluate the free energy of the receptor-lipid raft complex. We approximate the probability of finding the biomolecule at position ***r*** by: 
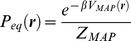
 where Z_MAP_ the canonical partition function 
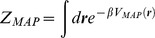
 and β = 1/k_B_T. The free energy can be evaluated directly: F = −1/βlog(Z_MAP_).

## Supporting Information

Video S1 Evolution of the confining potential acting on 4 receptors of the ε-toxin after the addition of 20 U/ml of cholesterol oxidase. The video starts after the addition of cholesterol oxidase. The video displays 5 images per second. The temporal inference window is 40 seconds and the window is shifted by 5 seconds between each frame. The potential is plotted on the points visited by the receptor.
(MP4)

Video S2 Evolution of the confining potential acting on 4 receptors of the ε-toxin after the addition of 10 U/ml of sphingomyelinase. The video starts after the addition of sphingomyelinase. The video displays 5 images per second. The temporal inference window is 40 seconds and the window is shifted by 5 seconds between each frame. The potential is plotted on the points visited by the receptor.
(MP4)

Video S3 Videoof the confined motion of a receptor, during which a hopping event takes place. A Pixel is 254 nm. The hopping event happens at 55 s.
(MP4)

Figure S1 Temporal evolution of the average diffusion in the raft (A) and spring constant (B) after the addition of cholesterol oxidase (in Black) and of sphingomyelinase (in Red).
(TIFF)

Figure S2 Temporal evolution of standard deviation of the diffusivity map after adding cholesterol oxidase (black) and after adding sphingomyelinase (red). Thin lines are individual experiments and thick lines are the average values of all individual experiments.
(TIFF)

Figure S3 Hopping in 1D between two wells. E_h_ is the hopping energy.
(TIFF)

Figure S4 Hopping in 1D between a confining well and free motion. E_h_ is the hopping energy.
(TIFF)

Figure S5 Hopping energy in 2D between two confining wells. The straight red line joins the two confining wells' minima (here chosen to lie along the x-axis). The hopping energy is defined as the energy difference between the maximum potential value along this line and the lowest potential minimum of the two wells (in this case, the minimum of the well on the right). The curved line shows another possible way to go from one well to the other.
(TIFF)

Figure S6 Hopping between a confining well and free motion. The hopping energy is defined as the energy difference between the average value of the potential on the green circle and the minimal value of the potential in the well. Here, the domain is circular.
(TIFF)

Figure S7 Pdf of the inferred hopping energy for the MAP estimator (Black) and for the average value of the *posteriori* probability distribution estimator (AVE, Red) for hopping between two confining wells in 2D. 2000 point trajectories, 3 k_B_T theoretical hopping energy (shown by the thick vertical black line), 200 nm between the two wells, 0.025 µm^2^·s^−1^ diffusivity and 25 ms acquisition time.
(TIFF)

Figure S8 Pdf of the MAP for the hopping energy between two confining wells in 2D in log scale. The red curve is the asymptotic Gaussian decay. 2000 points trajectories, 3 k_B_T theoretical hopping energy (shown by the thick vertical black line), 400 nm between the two wells, 0.025 µm^2^·s^−1^ diffusivity and 25 ms acquisition time.
(TIFF)

Figure S9 Evolution of the inferred diffusion coefficient (normalized to the no-noise limit) with the standard deviation of the potential noise for 1D double-well trajectories. The black dots are the average values of the MAP statistics and the red line is the Zwanzig model [5] that models the effect of potential noise on the diffusivity. 2000-point trajectories, 3 k_B_T theoretical hopping energy, 400 nm between the two wells, 0.025 µm^2^·s^−1^ diffusivity and 25 ms acquisition time.
(TIFF)

Figure S10 Evolution of the inferred diffusion coefficient (normalized to the no-noise limit) with the standard deviation of the potential noise for 2D double-well trajectories. The black dots are the average values of the MAP statistics and the red line is the 1D Zwanzig model [5]. 2000-point trajectories, 3 k_B_T theoretical hopping energy, harmonic confinement along the y-axis with spring constant 200 k_B_T·µm^−2^, 300 nm between the two wells, 0.035 µm^2^·s^−1^ diffusivity and 25 ms acquisition time.
(TIFF)

Figure S11 Evolution of the MAP Pdf with hopping energy for trajectories with a unique hopping event. 3 k_B_T hopping energy in black, 5 k_B_T hopping energy in red and 7.5 k_B_T hopping energy in green. 2000-point trajectories, 300 nm between the two wells, 0.025 µm^2^·s^−1^ diffusivity and 25 ms acquisition time.
(TIFF)

Figure S12 Evolution of the MAP Pdf with lateral confinement spring constant for k = 50 k_B_T·µm^−2^ (black), k = 100 k_B_T·µm^−2^ (red), k = 200 k_B_T·µm^−2^ (green), k = 250 k_B_T·µm^−2^ (blue). 2000-point trajectories, 3 k_B_T theoretical hopping energy (shown by the thick vertical black line), 0.035 µm^2^·s^−1^ diffusivity, 400 nm between the two wells, and 25 ms acquisition time.
(TIFF)

Figure S13 Evolution of the MAP Pdf of the hopping energy with the diffusivity for a 1D double-well potential. D = 0.01 µm^2^·s^−1^ in black, D = 0.02 µm^2^·s^−1^ in red, D = 0.04 µm^2^·s^−1^ in green, D = 0.075 µm^2^·s^−1^ in blue, D = 0.1 µm^2^·s^−1^ in cyan. 2000-point trajectories, 3 k_B_T theoretical hopping energy (vertical black line), 400 nm between the two wells, and 25 ms acquisition time.
(TIFF)

Figure S14 Evolution of the average MAP values with diffusivity. The black dots are the results of the inferences and the red line is a parabolic fit.
(TIFF)

Figure S15 Evolution of the MAP Pdf with varying central diffusivity for a 1D double-well potential. D = 0.00625 µm^2^·s^−1^ in black, D = 0.0125 µm^2^·s^−1^ in red, D = 0.05 µm^2^·s^−1^ in green, D = 0.075 µm^2^·s^−1^ in blue, D = 0.125 µm^2^·s^−1^ in cyan. 2000-point trajectories, 3 k_B_T theoretical hopping energy (shown by the thick vertical black line), diffusivities in the wells D = 0.025 µm^2^·s^−1^, 400 nm between the two wells, and 25 ms acquisition time.
(TIFF)

Figure S16 Evolution of the MAP Pdf with varying central diffusivity for a 2D double-well potential. D = 0.00625 µm^2^·s^−1^ in black, D = 0.0125 µm^2^·s^−1^ in red, D = 0.05 µm^2^·s^−1^ in green, D = 0.075 µm^2^·s^−1^ in blue, D = 0.125 µm^2^·s^−1^ in cyan. 2000-point trajectories, 3 k_B_T theoretical hopping energy (shown by the thick vertical black line), diffusivities in the wells D = 0.035 µm^2^·s^−1^, 400 nm between the two wells, and 25 ms acquisition time.
(TIFF)

Figure S17 Evolution of the MAP Pdf with the external diffusivity. D = 0.0088 µm^2^·s^−1^ in black, D = 0.0175 µm^2^. ·s^−1^ in red, D = 0.07 µm^2^·s^−1^ in green, D = 0.105 µm^2^·s^−1^ in blue, D = 0.175 µm^2^·s^−1^ in cyan and D = 0.35 µm^2^·s^−1^ in yellow. 2000-point trajectories with at least 1000 points inside the well, 3 k_B_T theoretical hopping energy (shown by the thick vertical black line), diffusivity inside the well D = 0.035 µm^2^·s^−1^, 100 nm radius of the well, and 25 ms acquisition time.
(TIFF)

Figure S18 Evolution of the MAP Pdf with external diffusivity. D = 0.0125 µm^2^·s^−1^ in black, D = 0.05 µm^2^·s^−1^ in red, D = 0.125 µm^2^·s^−1^ in green, and D = 0.25 µm^2^·s^−1^ in blue. 1000-point trajectories with at least 500 points in the well, 5 k_B_T theoretical hopping energy (shown by the thick vertical black line), diffusivity inside the well D = 0.025 µm^2^·s^−1^, 100 nm radius of the well, and 25 ms acquisition time.
(TIFF)

Figure S19 Evolution of the inferred value of the diffusivity (A) and spring constant (B) with the input diffusivity and spring constant used in the numerical simulations of the trajectories.
(TIFF)

Figure S20 Three examples of two-well potentials with low hopping energy between the two wells. On the left, only the trajectory points are superimposed on the image; on the right, the trajectory points are linked to materialize the trajectory. The inferred hopping energies are from top to bottom: 0.47, 0.26, and 0.43 k_B_T.
(TIFF)

Figure S21 Three examples of two-well potentials with high hopping energy between the two wells. On the left, only the trajectory points are superimposed on the image; on the right, the trajectory points are linked to materialize the trajectory. The inferred hopping energies are from top to bottom: 1.7, 1.2, and 2.3 k_B_T.
(TIFF)

Figure S22 Diffusivity and Potential Map with the full visible trajectory of the receptor corresponding to Figure 4 A) Diffusivity map of the membrane area where the receptor moves. The diffusivity field was generated by a bi-harmonic interpolation of the inferred diffusivity field on the mesh. B) Inferred interaction energy map felt by the receptor. Black lines connect the successive positions of the biomolecule.
(TIFF)

Figure S23 Interaction potential acting on the ε-toxin receptor inferred from a 4262-point trajectory. On the left, only the trajectory points are superimposed on the image; on the right, the trajectory points are linked to materialize the trajectory.
(TIFF)

Document S1 We expose various experimental results, discuss the definition of hopping events and quantify the efficiency of the inference.
(PDF)
