# Supplementary figures and images for: Probing Membrane Protein Interactions with Their Lipid Raft Environment Using Single-Molecule Tracking and Bayesian Inference Analysis
Source: PLoS One. 2013 Jan 3;8(1):e53073. doi: 10.1371/journal.pone.0053073 (PMC3536804; doi:10.1371/journal.pone.0053073)

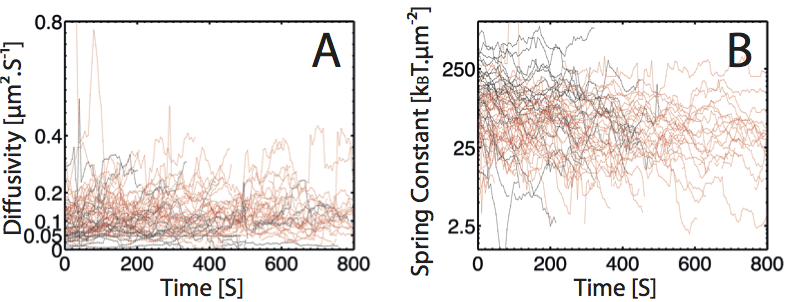

Supplement: Figure S1 — Temporal evolution of the average diffusion in the raft (A) and spring constant (B) after the addition of cholesterol oxidase (in Black) and of sphingomyelinase (in Red). (TIFF) [file pone.0053073.s004.tiff]

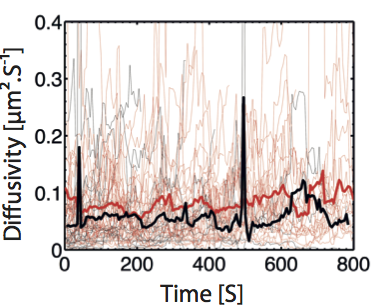

Supplement: Figure S2 — Temporal evolution of standard deviation of the diffusivity map after adding cholesterol oxidase (black) and after adding sphingomyelinase (red). Thin lines are individual experiments and thick lines are the average values of all individual experiments. (TIFF) [file pone.0053073.s005.tiff]

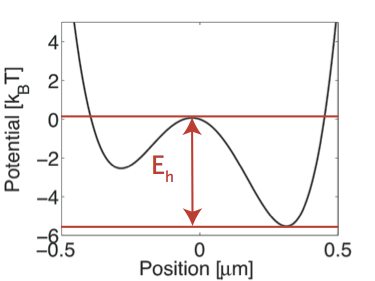

Supplement: Figure S3 — Hopping in 1D between two wells. Eh is the hopping energy. (TIFF) [file pone.0053073.s006.tiff]

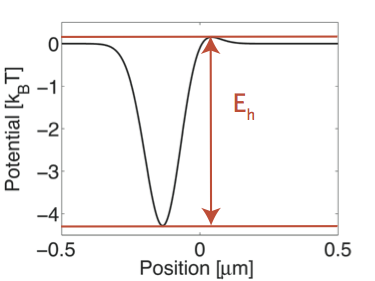

Supplement: Figure S4 — Hopping in 1D between a confining well and free motion. Eh is the hopping energy. (TIFF) [file pone.0053073.s007.tiff]

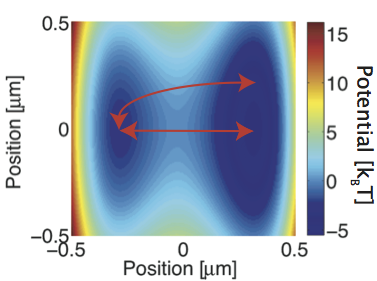

Supplement: Figure S5 — Hopping energy in 2D between two confining wells. The straight red line joins the two confining wells' minima (here chosen to lie along the x-axis). The hopping energy is defined as the energy difference between the maximum potential value along this line and the lowest potential minimum of the two wells (in this case, the minimum of the well on the right). The curved line shows another possible way to go from one well to the other. (TIFF) [file pone.0053073.s008.tiff]

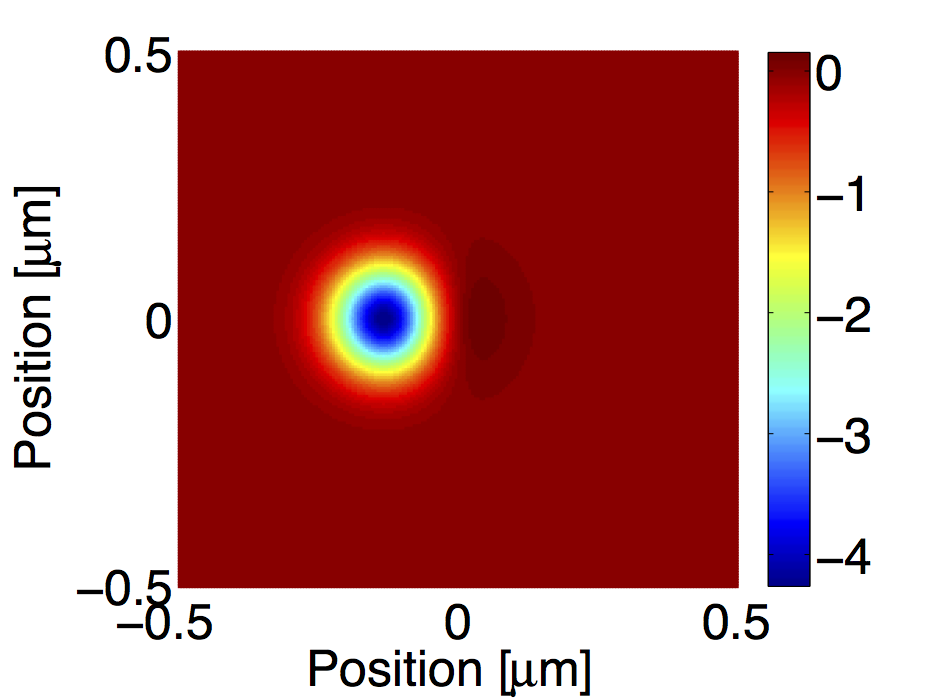

Supplement: Figure S6 — Hopping between a confining well and free motion. The hopping energy is defined as the energy difference between the average value of the potential on the green circle and the minimal value of the potential in the well. Here, the domain is circular. (TIFF) [file pone.0053073.s009.tiff]

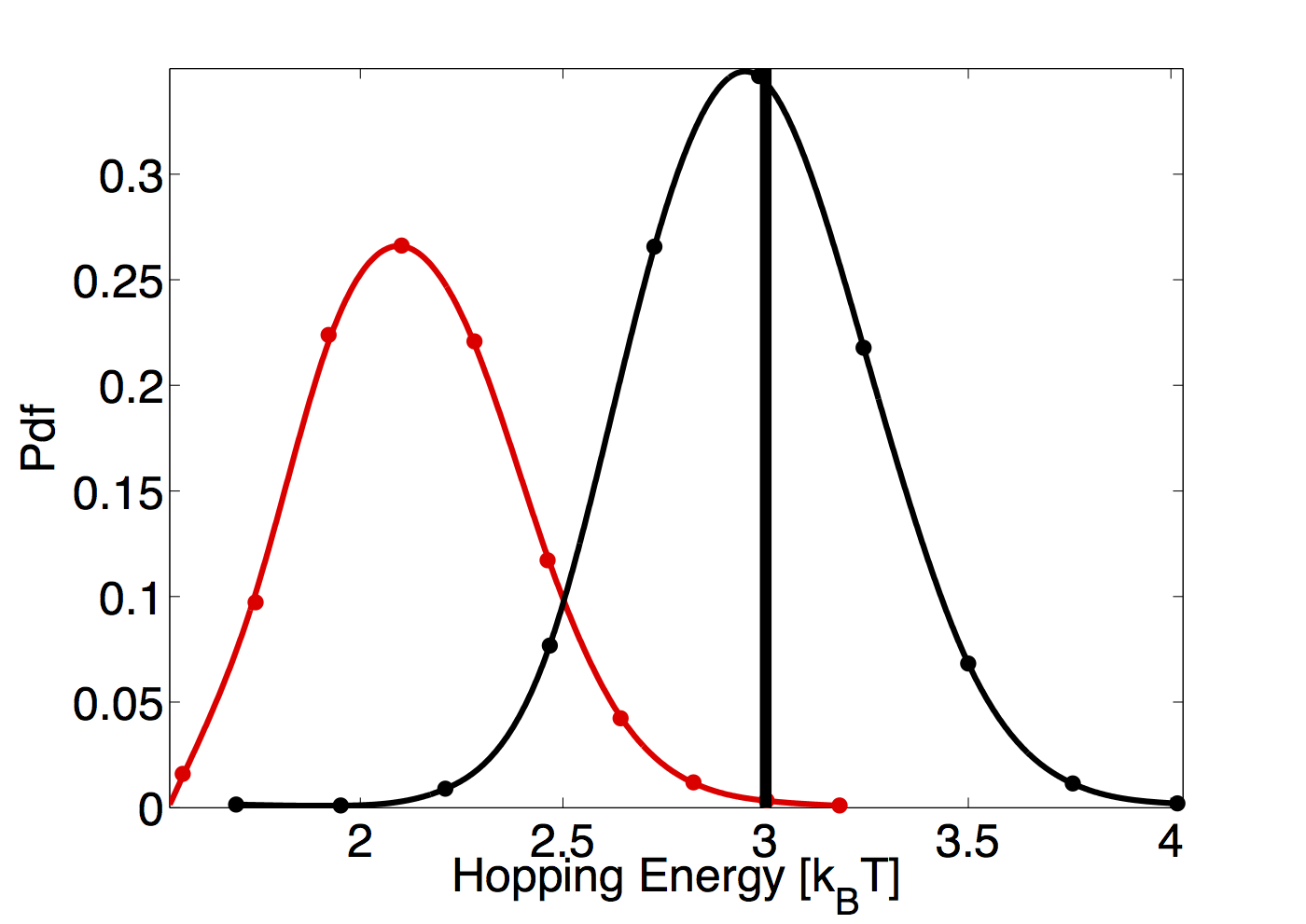

Supplement: Figure S7 — Pdf of the inferred hopping energy for the MAP estimator (Black) and for the average value of the posteriori probability distribution estimator (AVE, Red) for hopping between two confining wells in 2D. 2000 point trajectories, 3 kBT theoretical hopping energy (shown by the thick vertical black line), 200 nm between the two wells, 0.025 µm2·s−1 diffusivity and 25 ms acquisition time. (TIFF) [file pone.0053073.s010.tiff]

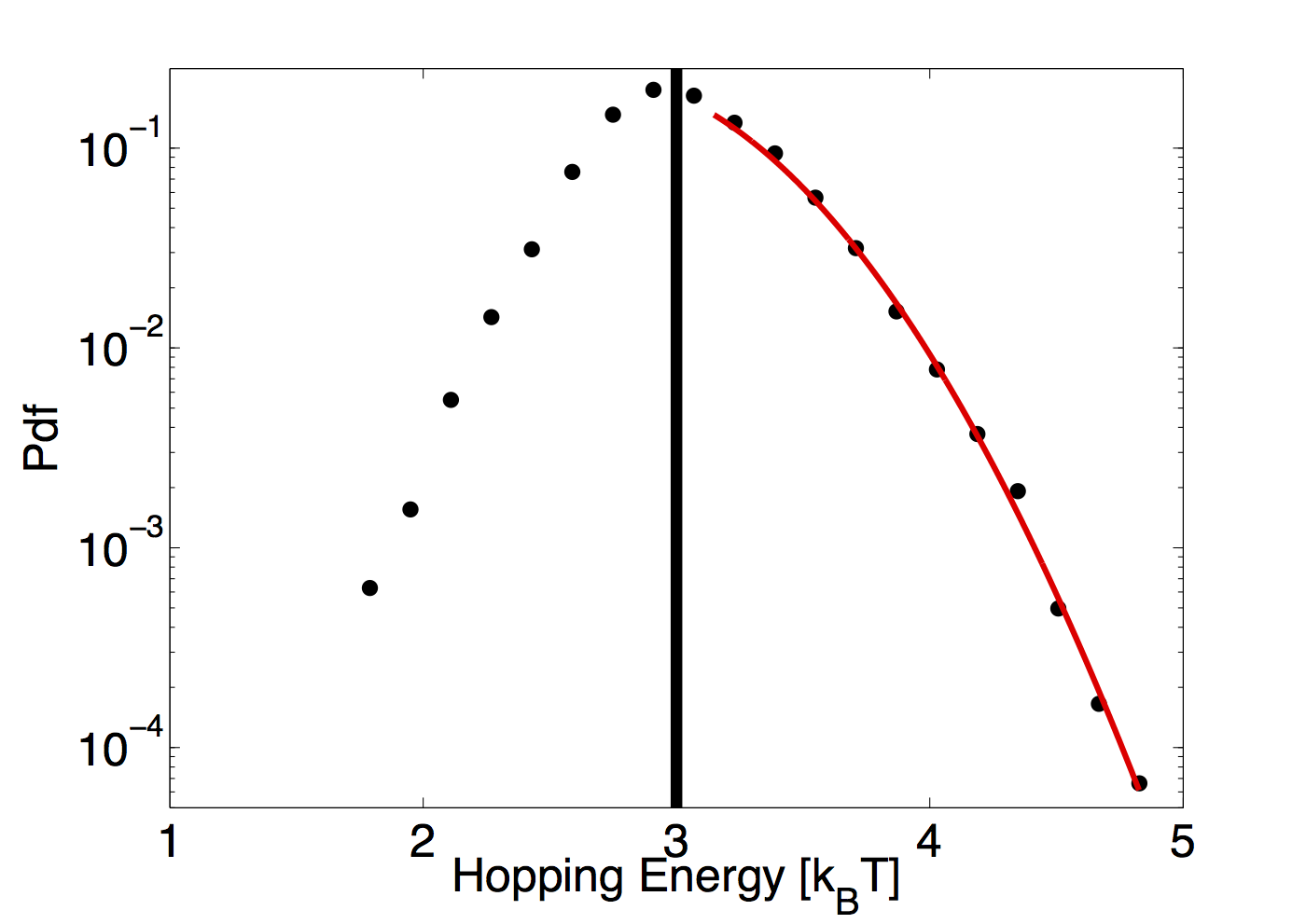

Supplement: Figure S8 — Pdf of the MAP for the hopping energy between two confining wells in 2D in log scale. The red curve is the asymptotic Gaussian decay. 2000 points trajectories, 3 kBT theoretical hopping energy (shown by the thick vertical black line), 400 nm between the two wells, 0.025 µm2·s−1 diffusivity and 25 ms acquisition time. (TIFF) [file pone.0053073.s011.tiff]

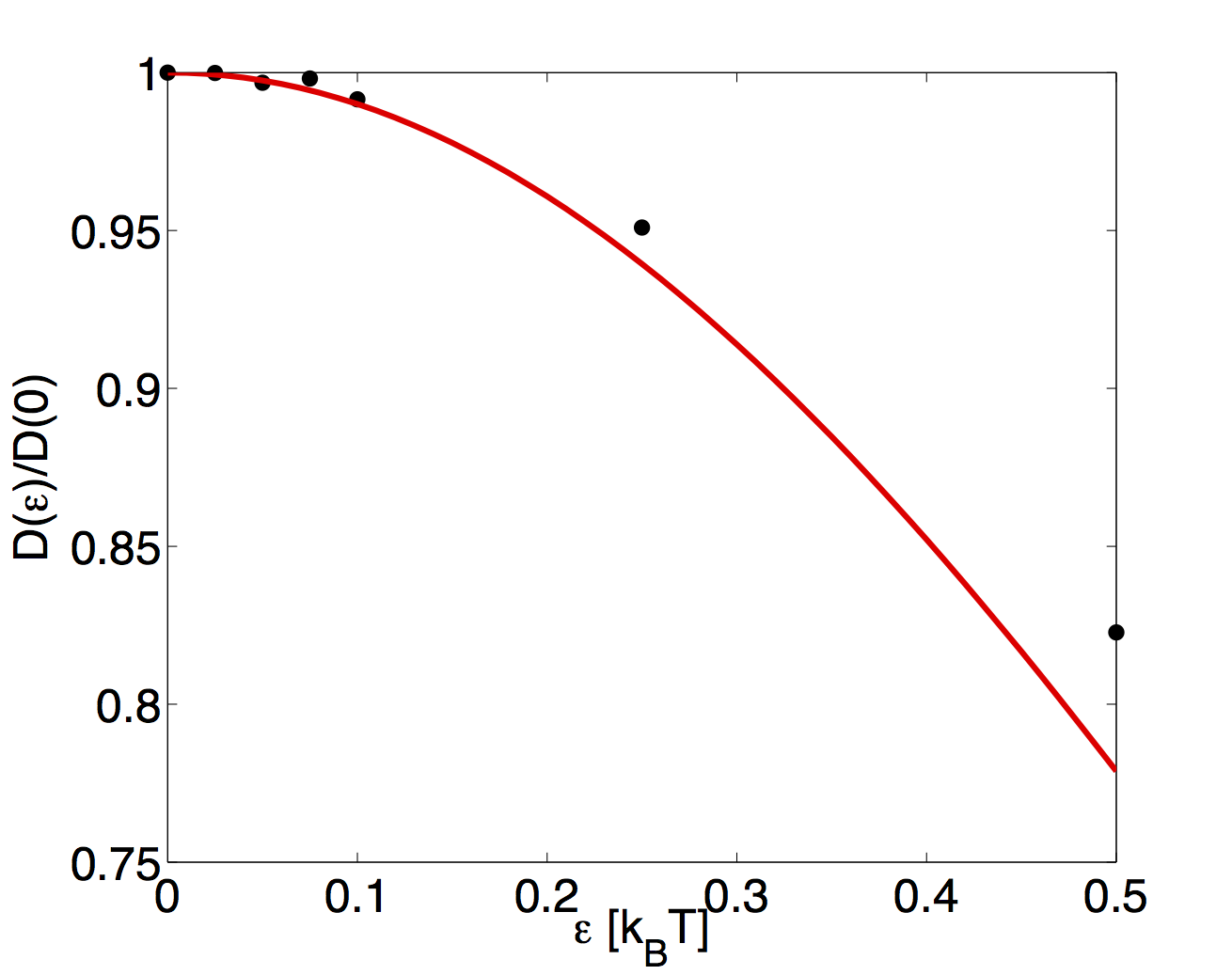

Supplement: Figure S9 — Evolution of the inferred diffusion coefficient (normalized to the no-noise limit) with the standard deviation of the potential noise for 1D double-well trajectories. The black dots are the average values of the MAP statistics and the red line is the Zwanzig model [5] that models the effect of potential noise on the diffusivity. 2000-point trajectories, 3 kBT theoretical hopping energy, 400 nm between the two wells, 0.025 µm2·s−1 diffusivity and 25 ms acquisition time. (TIFF) [file pone.0053073.s012.tiff]

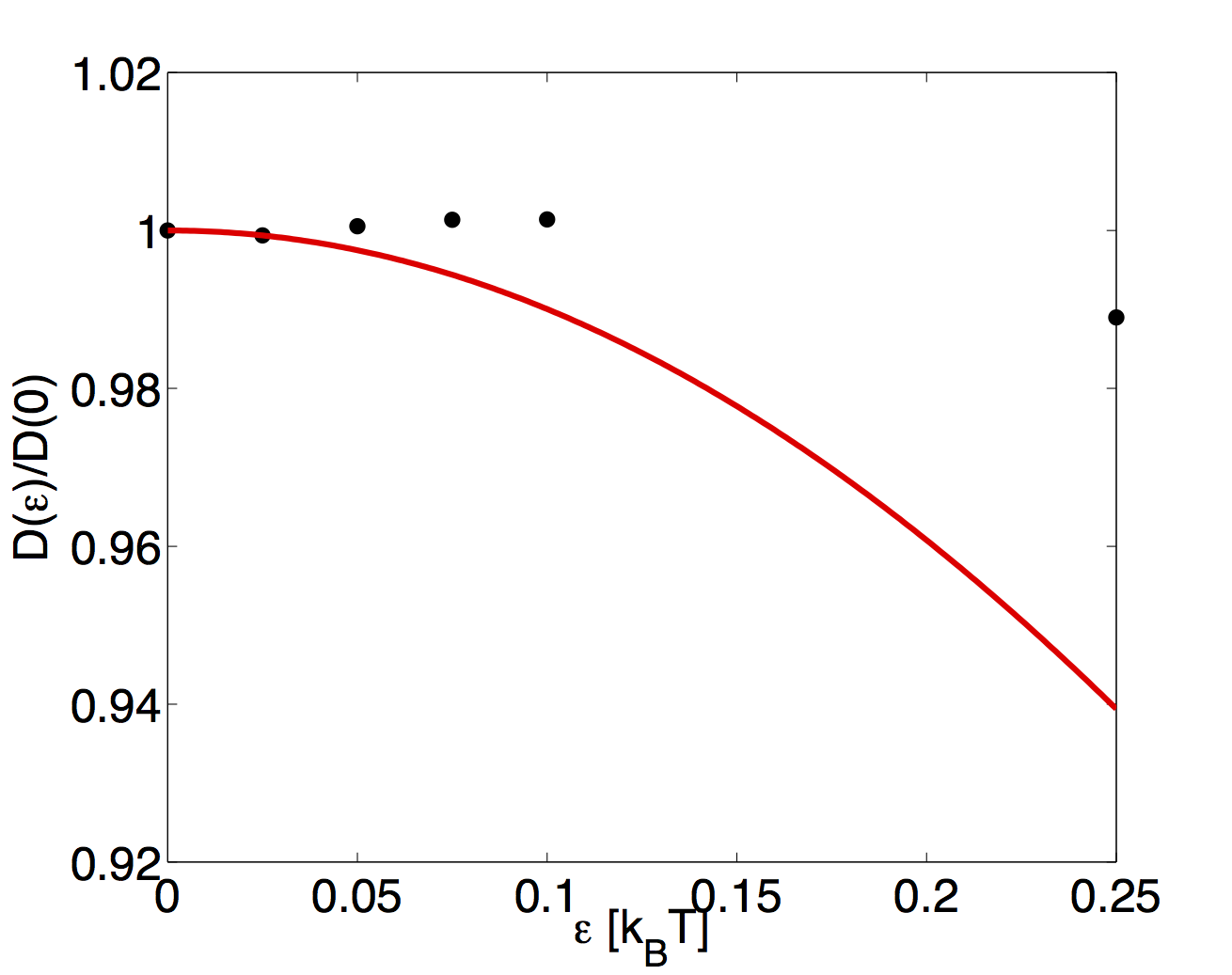

Supplement: Figure S10 — Evolution of the inferred diffusion coefficient (normalized to the no-noise limit) with the standard deviation of the potential noise for 2D double-well trajectories. The black dots are the average values of the MAP statistics and the red line is the 1D Zwanzig model [5]. 2000-point trajectories, 3 kBT theoretical hopping energy, harmonic confinement along the y-axis with spring constant 200 kBT·µm−2, 300 nm between the two wells, 0.035 µm2·s−1 diffusivity and 25 ms acquisition time. (TIFF) [file pone.0053073.s013.tiff]

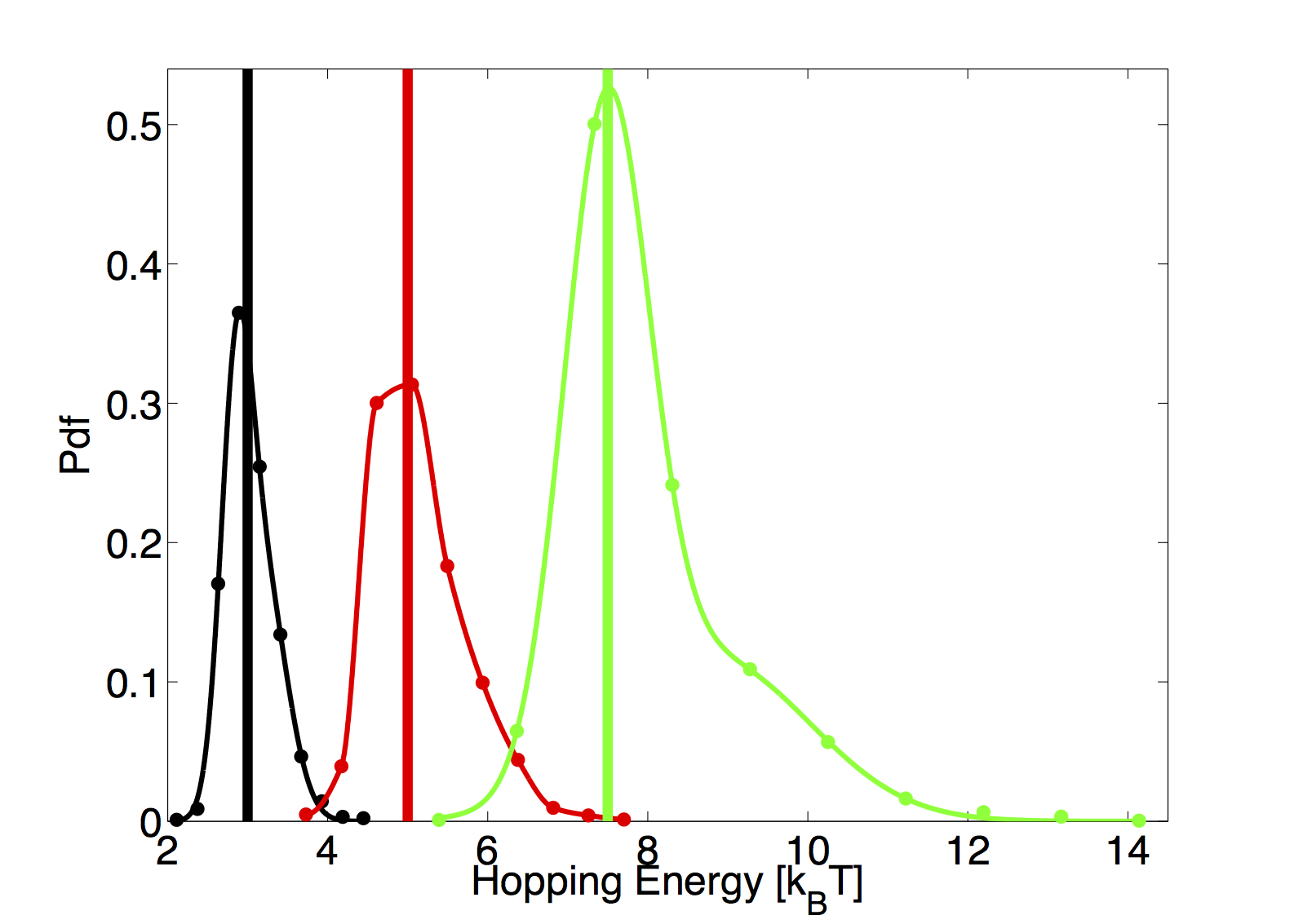

Supplement: Figure S11 — Evolution of the MAP Pdf with hopping energy for trajectories with a unique hopping event. 3 kBT hopping energy in black, 5 kBT hopping energy in red and 7.5 kBT hopping energy in green. 2000-point trajectories, 300 nm between the two wells, 0.025 µm2·s−1 diffusivity and 25 ms acquisition time. (TIFF) [file pone.0053073.s014.tiff]

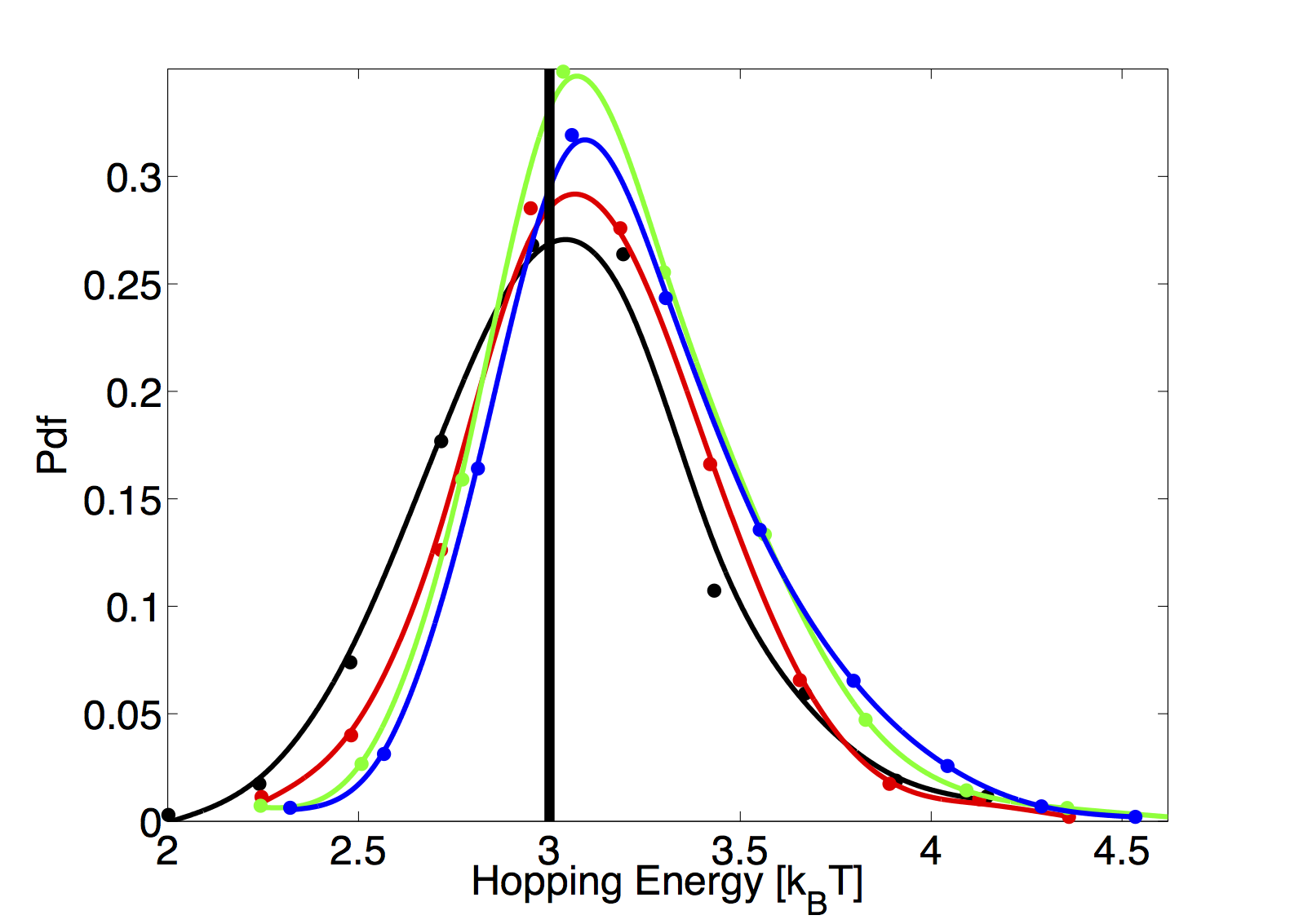

Supplement: Figure S12 — Evolution of the MAP Pdf with lateral confinement spring constant for k = 50 kBT·µm−2 (black), k = 100 kBT·µm−2 (red), k = 200 kBT·µm−2 (green), k = 250 kBT·µm−2 (blue). 2000-point trajectories, 3 kBT theoretical hopping energy (shown by the thick vertical black line), 0.035 µm2·s−1 diffusivity, 400 nm between the two wells, and 25 ms acquisition time. (TIFF) [file pone.0053073.s015.tiff]

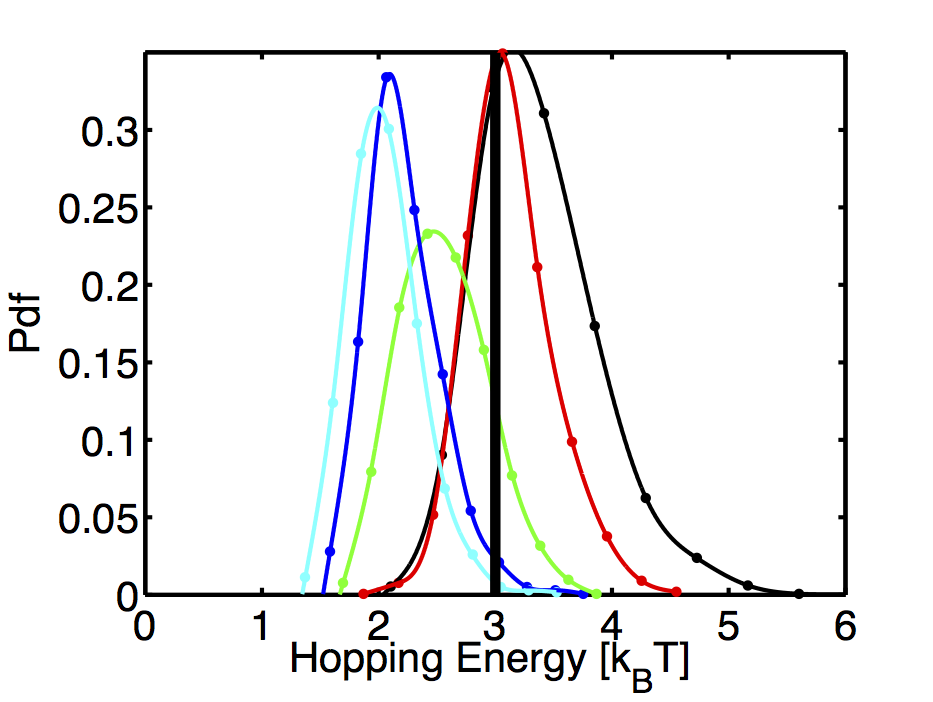

Supplement: Figure S13 — Evolution of the MAP Pdf of the hopping energy with the diffusivity for a 1D double-well potential. D = 0.01 µm2·s−1 in black, D = 0.02 µm2·s−1 in red, D = 0.04 µm2·s−1 in green, D = 0.075 µm2·s−1 in blue, D = 0.1 µm2·s−1 in cyan. 2000-point trajectories, 3 kBT theoretical hopping energy (vertical black line), 400 nm between the two wells, and 25 ms acquisition time. (TIFF) [file pone.0053073.s016.tiff]

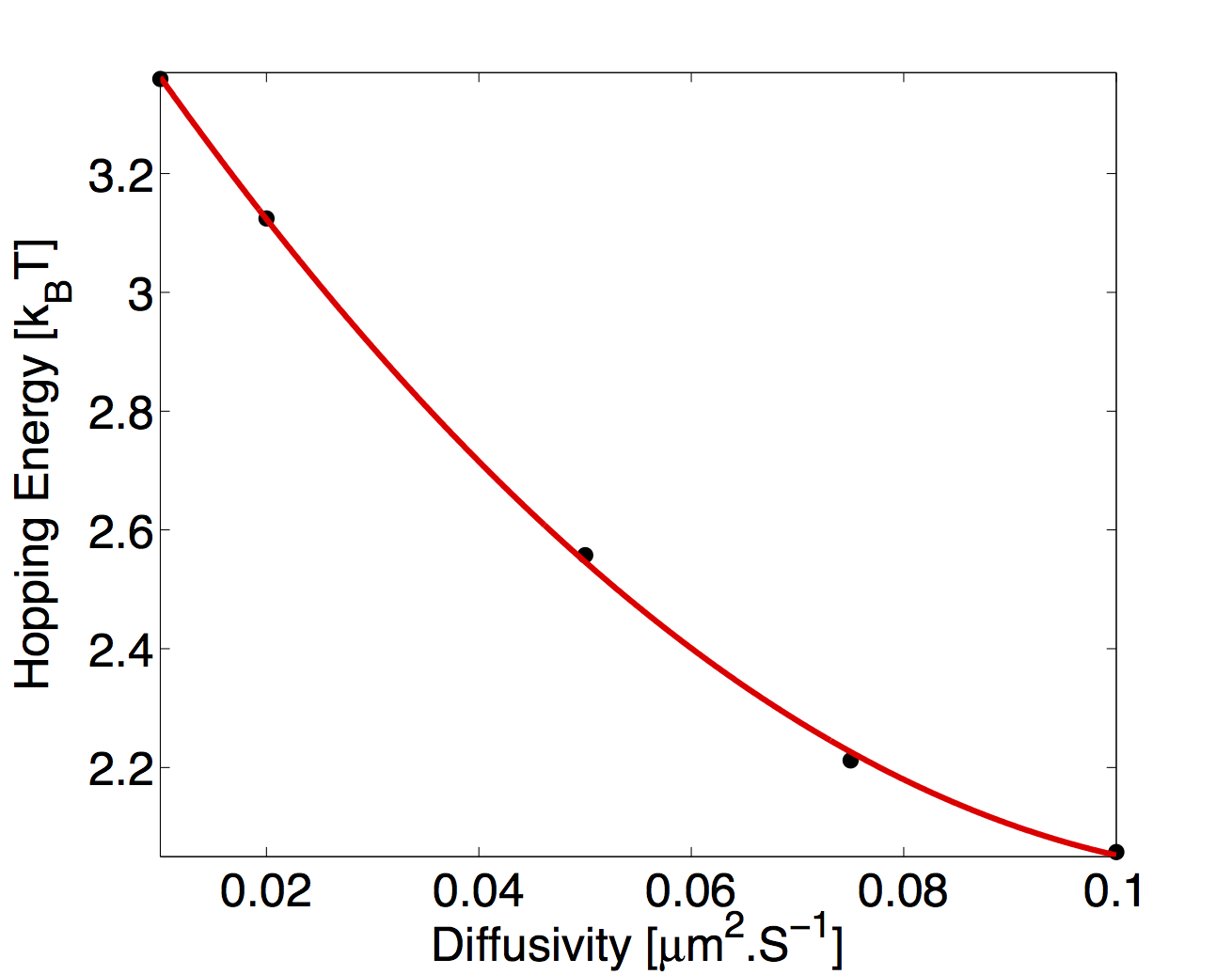

Supplement: Figure S14 — Evolution of the average MAP values with diffusivity. The black dots are the results of the inferences and the red line is a parabolic fit. (TIFF) [file pone.0053073.s017.tiff]

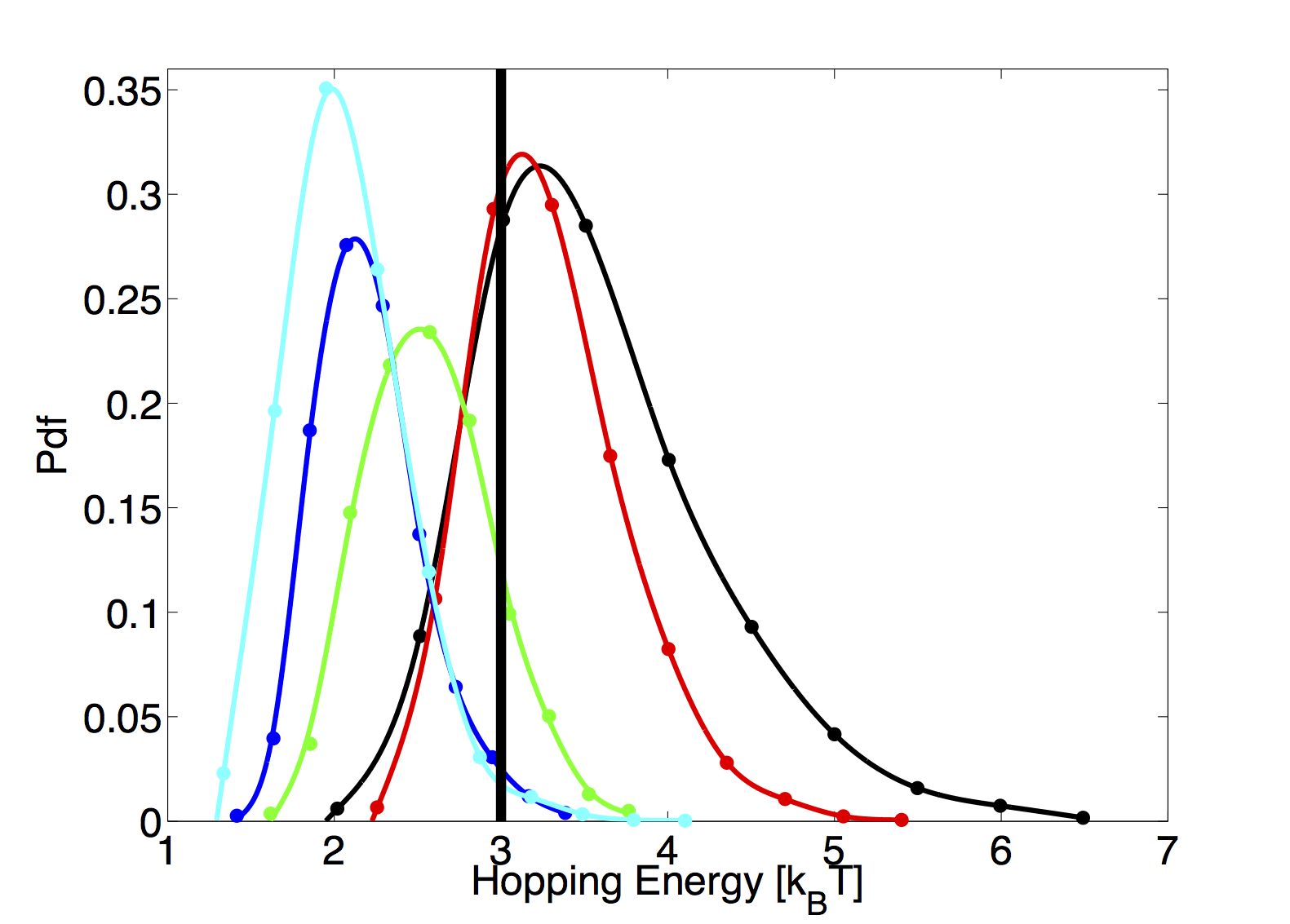

Supplement: Figure S15 — Evolution of the MAP Pdf with varying central diffusivity for a 1D double-well potential. D = 0.00625 µm2·s−1 in black, D = 0.0125 µm2·s−1 in red, D = 0.05 µm2·s−1 in green, D = 0.075 µm2·s−1 in blue, D = 0.125 µm2·s−1 in cyan. 2000-point trajectories, 3 kBT theoretical hopping energy (shown by the thick vertical black line), diffusivities in the wells D = 0.025 µm2·s−1, 400 nm between the two wells, and 25 ms acquisition time. (TIFF) [file pone.0053073.s018.tiff]

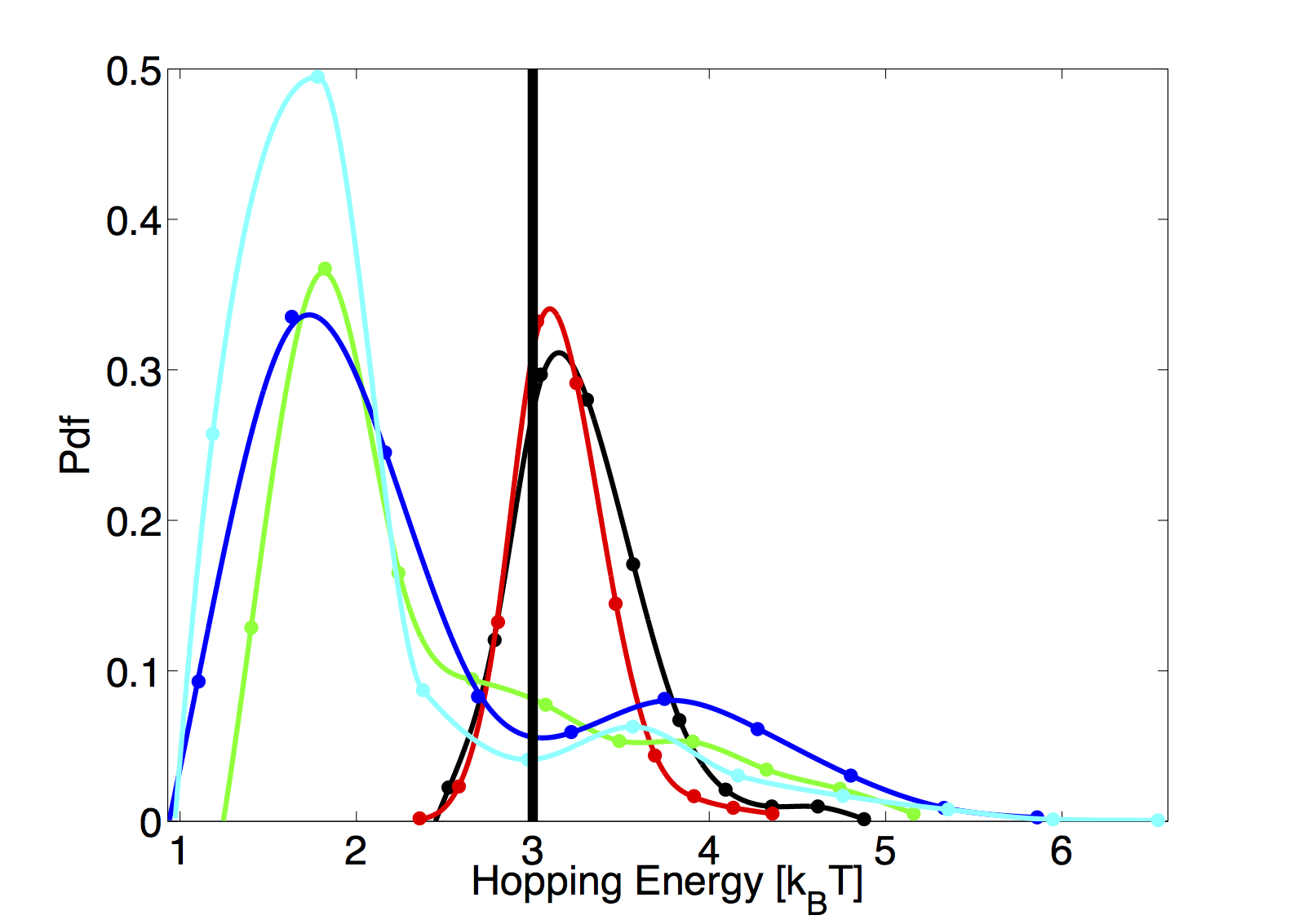

Supplement: Figure S16 — Evolution of the MAP Pdf with varying central diffusivity for a 2D double-well potential. D = 0.00625 µm2·s−1 in black, D = 0.0125 µm2·s−1 in red, D = 0.05 µm2·s−1 in green, D = 0.075 µm2·s−1 in blue, D = 0.125 µm2·s−1 in cyan. 2000-point trajectories, 3 kBT theoretical hopping energy (shown by the thick vertical black line), diffusivities in the wells D = 0.035 µm2·s−1, 400 nm between the two wells, and 25 ms acquisition time. (TIFF) [file pone.0053073.s019.tiff]

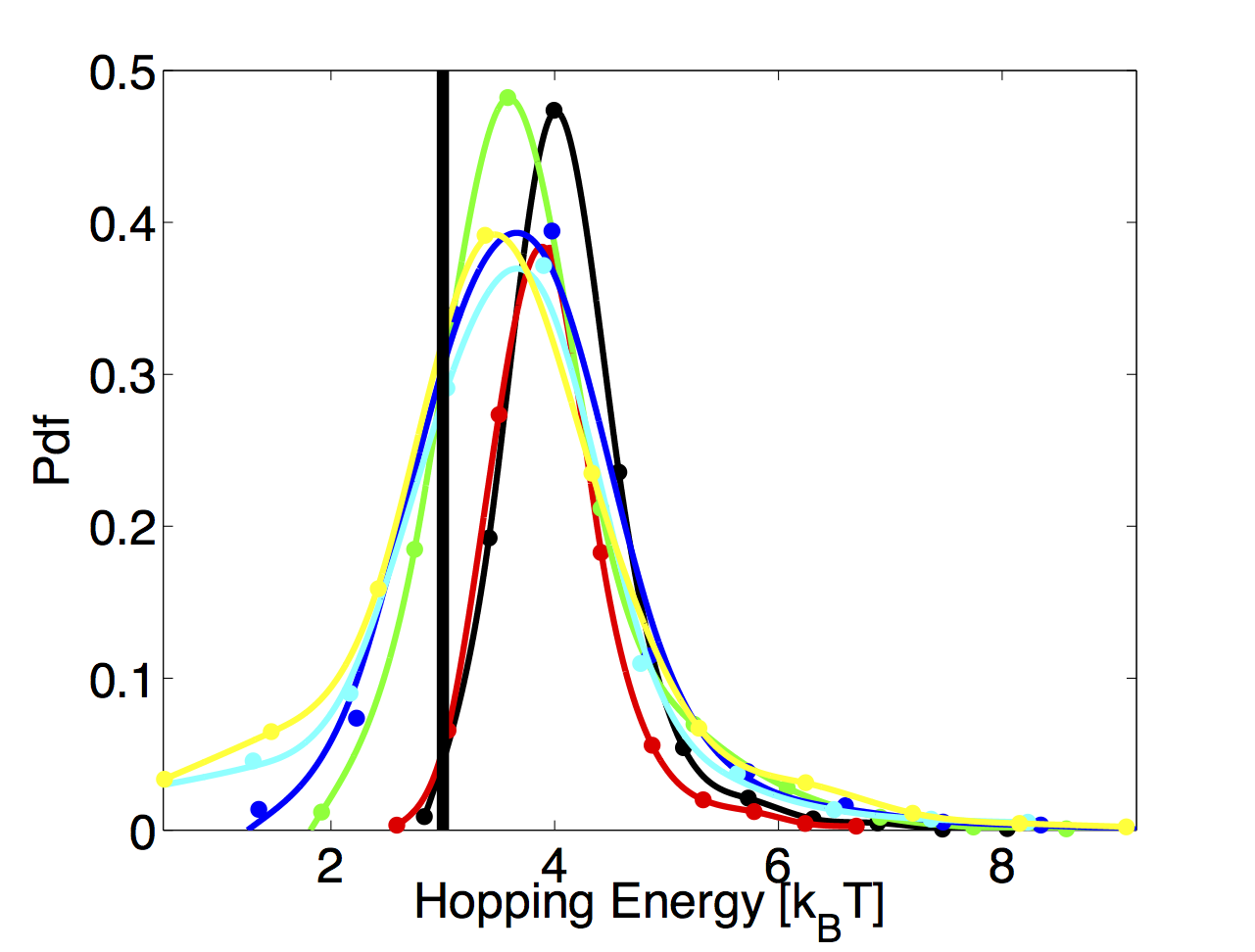

Supplement: Figure S17 — Evolution of the MAP Pdf with the external diffusivity. D = 0.0088 µm2·s−1 in black, D = 0.0175 µm2. ·s−1 in red, D = 0.07 µm2·s−1 in green, D = 0.105 µm2·s−1 in blue, D = 0.175 µm2·s−1 in cyan and D = 0.35 µm2·s−1 in yellow. 2000-point trajectories with at least 1000 points inside the well, 3 kBT theoretical hopping energy (shown by the thick vertical black line), diffusivity inside the well D = 0.035 µm2·s−1, 100 nm radius of the well, and 25 ms acquisition time. (TIFF) [file pone.0053073.s020.tiff]

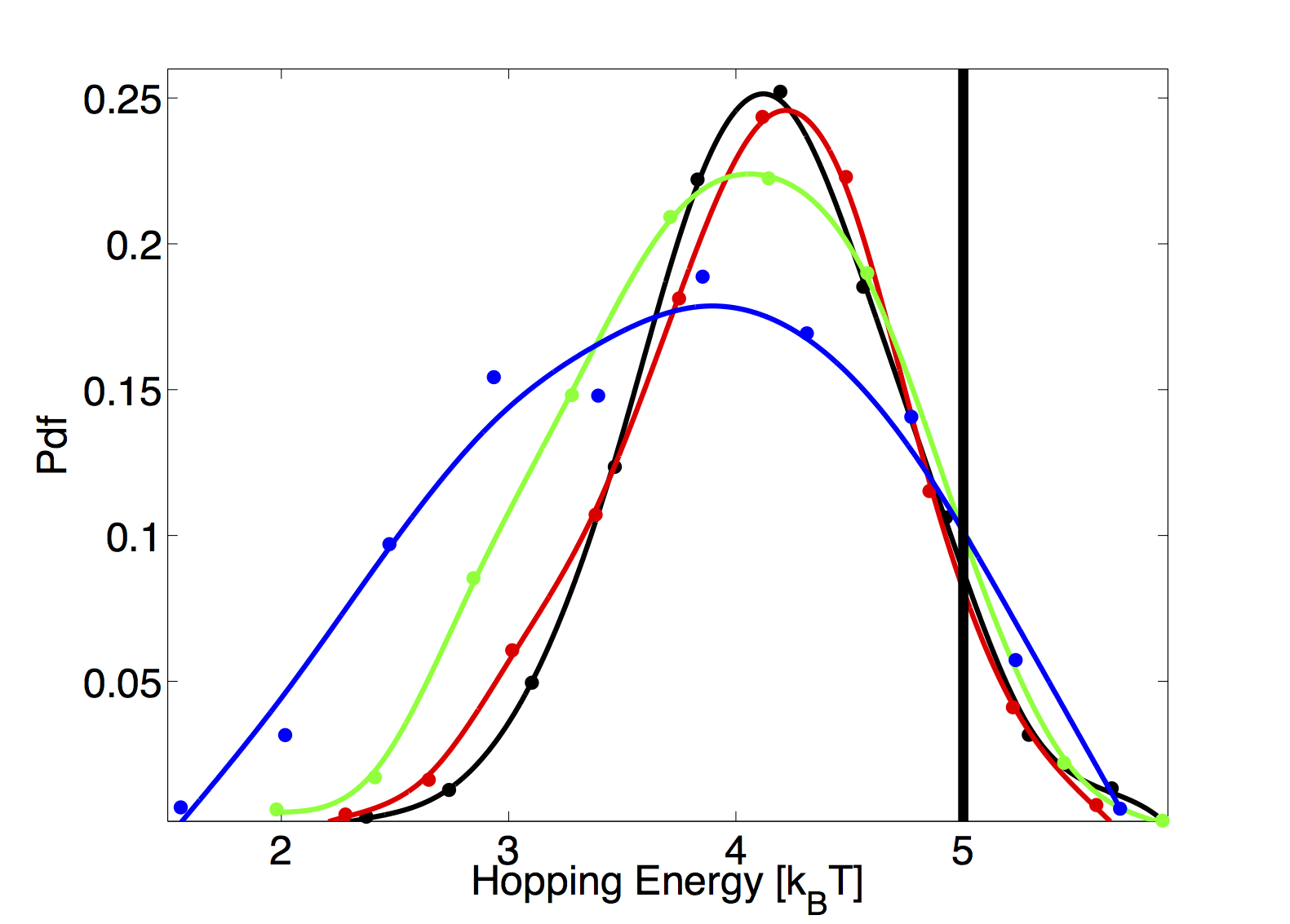

Supplement: Figure S18 — Evolution of the MAP Pdf with external diffusivity. D = 0.0125 µm2·s−1 in black, D = 0.05 µm2·s−1 in red, D = 0.125 µm2·s−1 in green, and D = 0.25 µm2·s−1 in blue. 1000-point trajectories with at least 500 points in the well, 5 kBT theoretical hopping energy (shown by the thick vertical black line), diffusivity inside the well D = 0.025 µm2·s−1, 100 nm radius of the well, and 25 ms acquisition time. (TIFF) [file pone.0053073.s021.tiff]

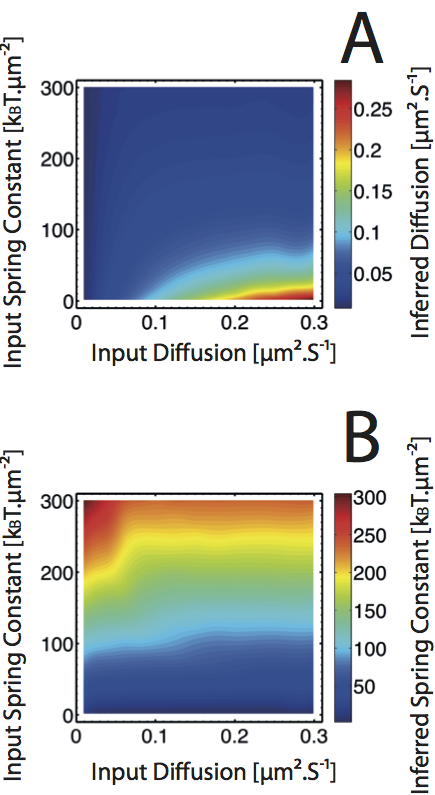

Supplement: Figure S19 — Evolution of the inferred value of the diffusivity (A) and spring constant (B) with the input diffusivity and spring constant used in the numerical simulations of the trajectories. (TIFF) [file pone.0053073.s022.tiff]

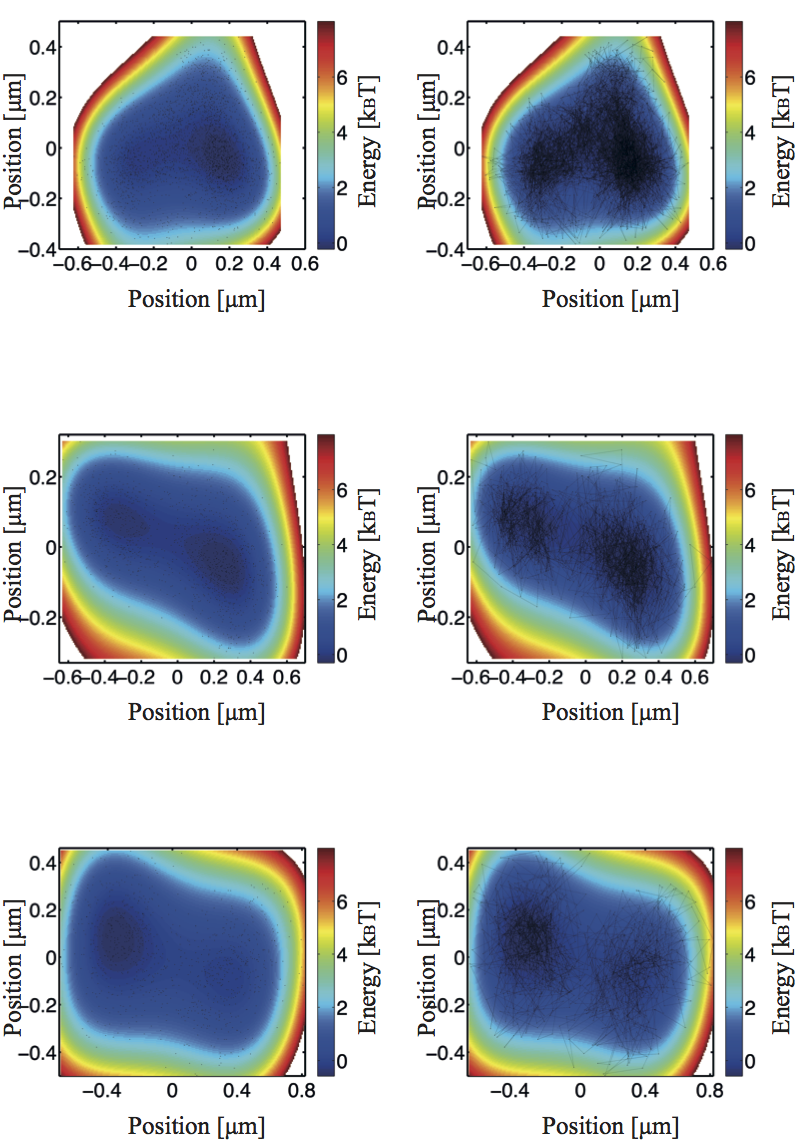

Supplement: Figure S20 — Three examples of two-well potentials with low hopping energy between the two wells. On the left, only the trajectory points are superimposed on the image; on the right, the trajectory points are linked to materialize the trajectory. The inferred hopping energies are from top to bottom: 0.47, 0.26, and 0.43 kBT. (TIFF) [file pone.0053073.s023.tiff]

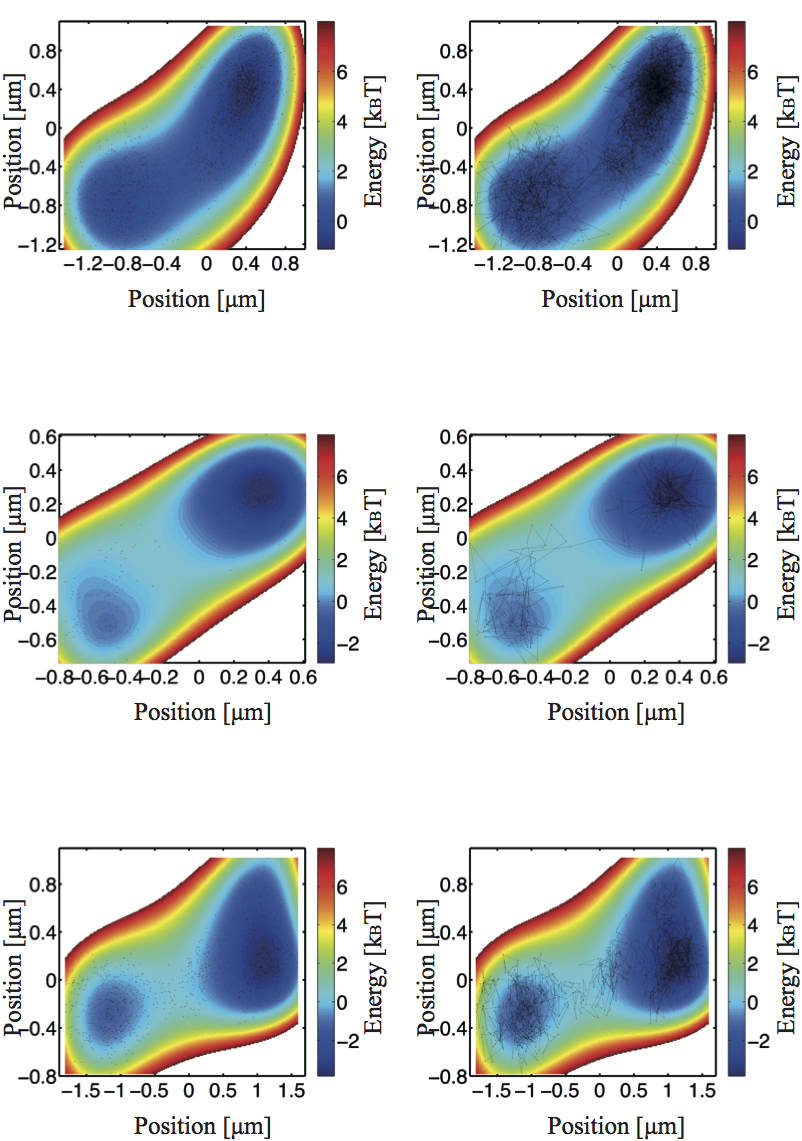

Supplement: Figure S21 — Three examples of two-well potentials with high hopping energy between the two wells. On the left, only the trajectory points are superimposed on the image; on the right, the trajectory points are linked to materialize the trajectory. The inferred hopping energies are from top to bottom: 1.7, 1.2, and 2.3 kBT. (TIFF) [file pone.0053073.s024.tif]

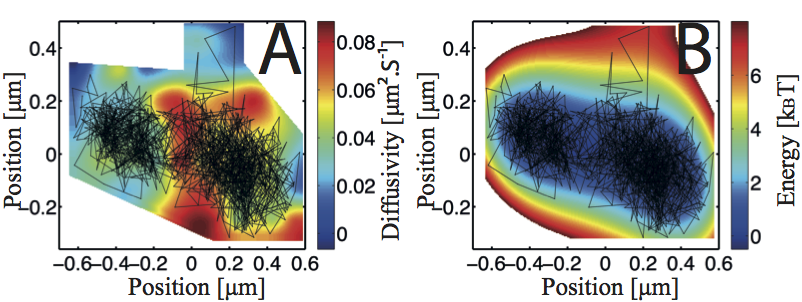

Supplement: Figure S22 — Diffusivity and Potential Map with the full visible trajectory of the receptor corresponding to Figure 4 A) Diffusivity map of the membrane area where the receptor moves. The diffusivity field was generated by a bi-harmonic interpolation of the inferred diffusivity field on the mesh. B) Inferred interaction energy map felt by the receptor. Black lines connect the successive positions of the biomolecule. (TIFF) [file pone.0053073.s025.tiff]

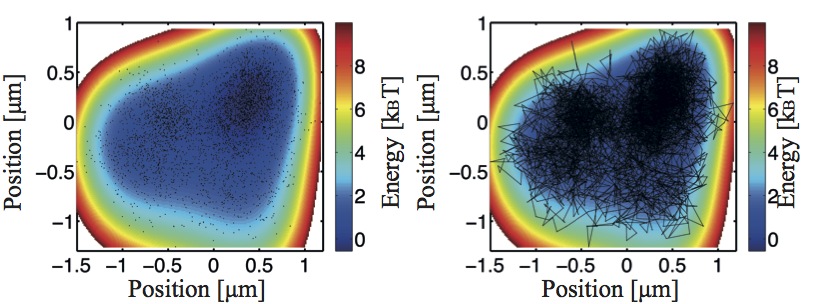

Supplement: Figure S23 — Interaction potential acting on the ε-toxin receptor inferred from a 4262-point trajectory. On the left, only the trajectory points are superimposed on the image; on the right, the trajectory points are linked to materialize the trajectory. (TIFF) [file pone.0053073.s026.tiff]
