# Supplementary material for: Probing Membrane Protein Interactions with Their Lipid Raft Environment Using Single-Molecule Tracking and Bayesian Inference Analysis
Source: PLoS One. 2013 Jan 3;8(1):e53073. doi: 10.1371/journal.pone.0053073 (PMC3536804; doi:10.1371/journal.pone.0053073)
Supplement: Document S1 — We expose various experimental results, discuss the definition of hopping events and quantify the efficiency of the inference. (PDF) [file pone.0053073.s027.pdf]

Supporting Information

Probing membrane protein interactions with their lipid raft environment using single molecule tracking and Bayesian inference analysis

Silvan Türkcan,[1,2,3] Maximilian U. Richly,[1] Antigoni Alexandrou,[1*] Jean-Baptiste Masson[2,3*]

[1]Laboratoire d'Optique et Biosciences, Ecole Polytechnique, CNRS, INSERM U696, 91128 Palaiseau Cedex, France
[2]Institut Pasteur, Physics of Biological Systems, 28 rue du Dr Roux, 75724 Paris Cedex 15, France
[3]CNRS, URA 2171, F-75015 Paris, France
*Corresponding authors: jbmasson@pasteur.fr, antigoni.alexandrou@polytechnique.edu

A. Evolution of the potentials with time

A.1 Videos

The video videoS1.avi displays the evolution of the confining potential acting on 4 receptors of the ε-toxin after the addition of 20 U/ml of cholesterol oxidase. The video starts after the addition of cholesterol oxidase. The video displays 5 images per second. The temporal inference window is 40 seconds and the window is shifted by 5 seconds between each frame. The potential is plotted on the points visited by the receptor.

The video videoS2.avi displays the evolution of the confining potential acting on 4 receptors of the ε-toxin after the addition of 10 U/ml of sphingomyelinase. The video starts after the addition of sphingomyelinase. The video displays 5 images per second. The temporal inference window is 40 seconds and the window is shifted by 5 seconds between each frame. The potential is plotted on the points visited by the receptor.

The video videoS3.avi shows the confined motion of a receptor, during which a hopping event takes place. A Pixel is 254 nm. The hopping event happens at 55 s.

A.2 Temporal evolution of the diffusivity and spring constant in the rafts

Here we give the temporal evolution of the average diffusion and of the spring constant (by approximating the potential fields by harmonic ones) after the addition of sphingomyelinase and cholesterol oxidase corresponding to figure S6.

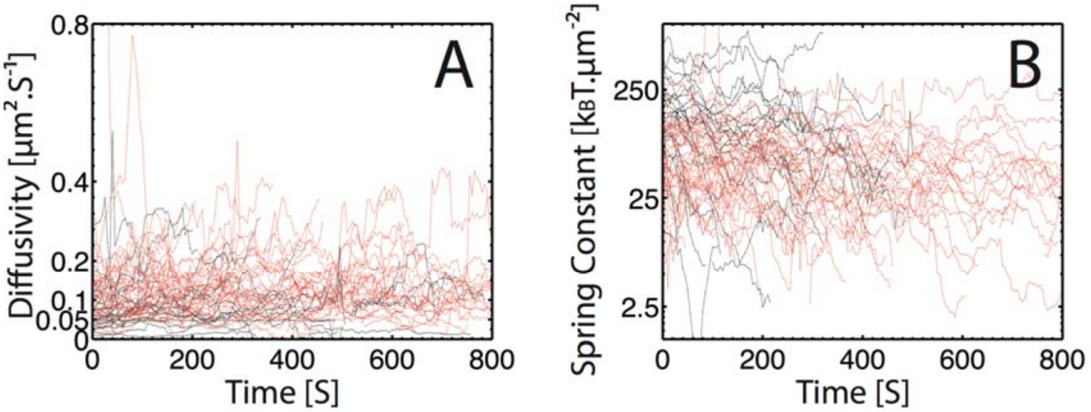

Figure S1: Temporal evolution of the average diffusion in the raft (A) and spring constant (B) after the adjunction of cholesterol oxidase (in black) and of sphingomyelinase (in red).

We also quantified the dispersion of diffusivity values inside the raft after the addition of sphingomyelinase and cholesterol oxidase (Fig. S2).

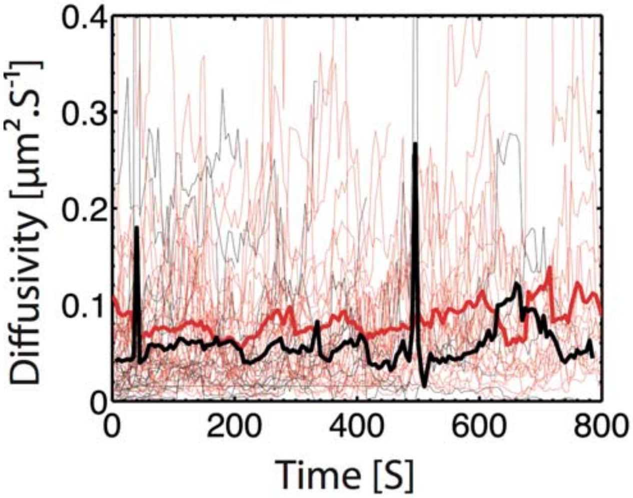

Figure S2: Temporal evolution of standard deviation of the diffusivity map after adding cholesterol oxidase (black) and after adding sphingomyelinase (red). Thin lines are individual experiments and thick lines are the average values of all individual experiments.

B.Definition of the hopping energy

Depending on the biological system and on its dynamics there is more than one way to define the hopping energy. Note that biological systems may exist where the hopping energy is not really definable. Here, we deal with confined motion in the membrane with escaping events;

hence, the hopping energy is the energy that the random walker needs to escape its confinement.

B.1 1D hopping between two wells

1D hopping between two wells is a well-defined system. We define the hopping energy to be equal to the energy difference between the deepest well and the maximum of the potential energy between the two wells.

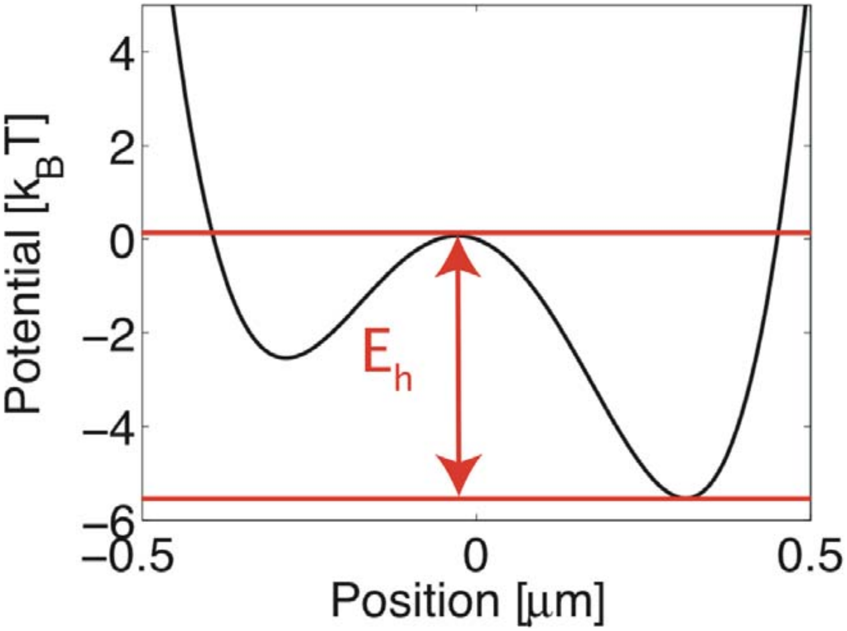

Figure S3: Hopping in 1D between two wells. E_h is the hopping energy.

B.2 1D hopping between a confining well and free motion

We define the hopping energy to be the energy difference between the maximal energy at the border of the domain and the minimal energy inside the confining domain.

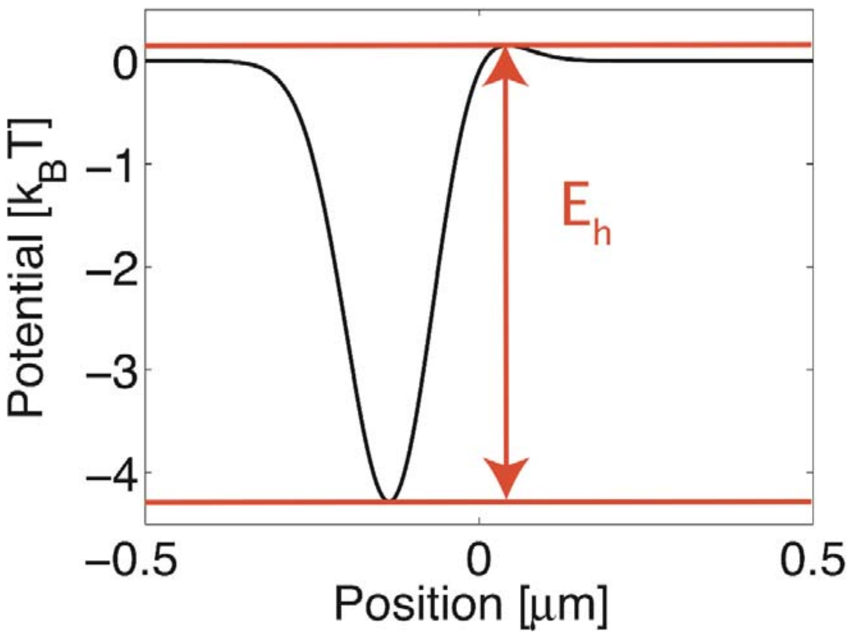

Figure S4: Hopping in 1D between a confining well and free motion. E_h is the hopping energy.

B.3 2D hopping between two wells

2D hopping between two confining wells leads to more issues in the definition of the hopping energy. Because of the multiple ways to access the second well (see Fig. S5), the definition of the hopping energy is not unique. We here define the hopping energy as the energy difference between the maximal potential value on the straight line joining the two confining well minima and the minimum value of the deepest potential well.

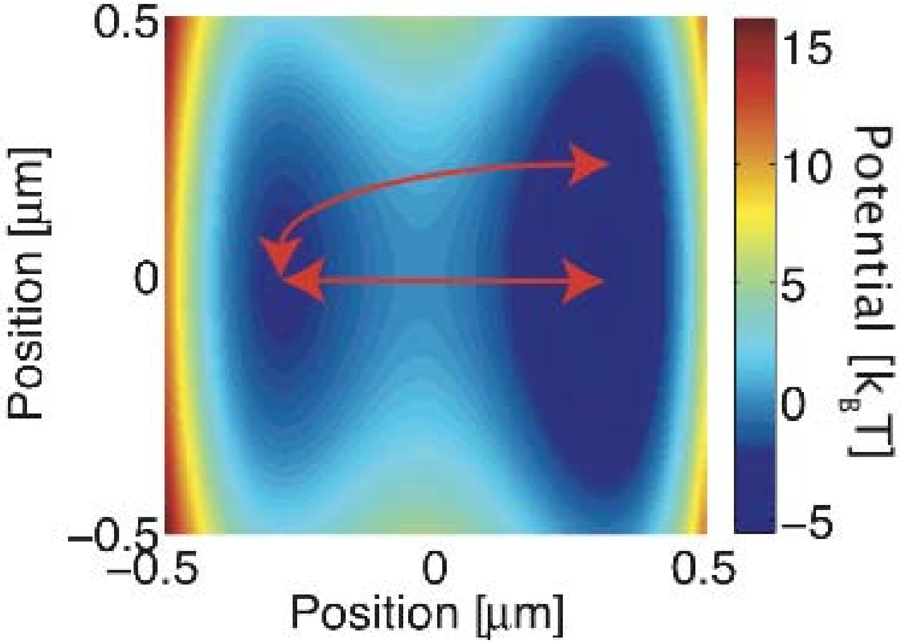

Figure S5: Hopping energy in 2D between two confining wells. The straight red line joins the two confining wells' minima (here chosen to lie along the x-axis). The hopping energy is defined as the energy difference between the maximum potential value along this line and the lowest potential minimum of the two wells (in this case, the minimum of the well on the right). The curved line shows another possible way to go from one well to the other.

B.4 2D hopping between a confining well and free motion

Here, the definition of the hopping energy is even more ambiguous because there are infinite ways to escape the confining area. We choose to define the hopping energy as the energy difference between the average value of the potential at the limit of the domain and the minimal value of the potential in the confining well (In the simulations the limit of the domains are known, in experimental work different definitions have to be devised depending on the nature of the confinement). This definition ignores the fact that, statistically, the biomolecule will tend to escape the well where the difference of potential is the lowest. Yet, simulations have shown that this definition is useful and it is also applied in Ref. [2] to study the confining potentials and hopping energies of the glycine receptors interacting with the scaffolding molecule gephyrin.

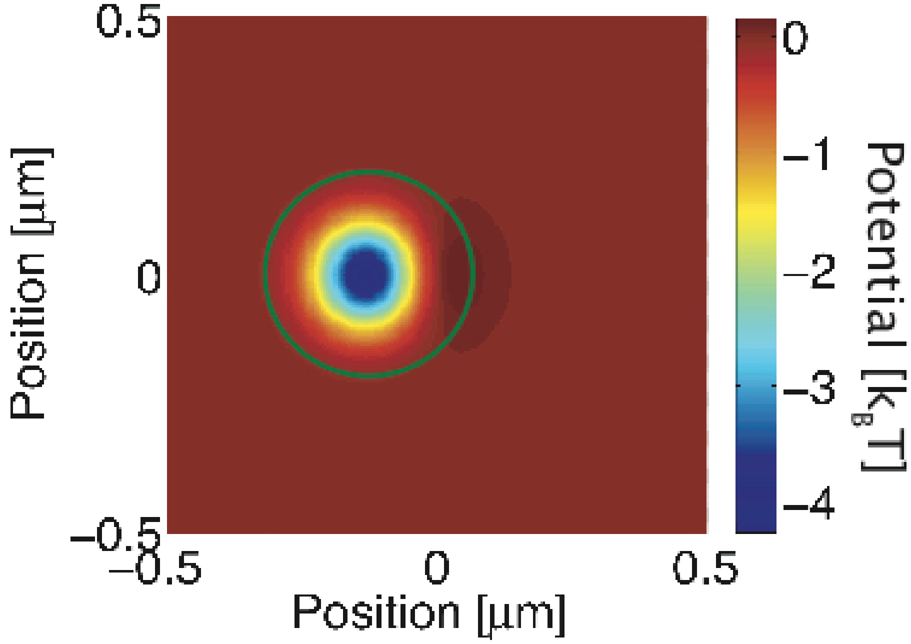

Figure S6: Hopping between a confining well and free motion. The hopping energy is defined as the energy difference between the average value of the potential on the green circle and the minimal value of the potential in the well. Here, the domain is circular.

C. Performance of the inference

The large variety of biologically relevant situations prevents an analytical evaluation of all possible outcomes of the inference scheme. Simulations of biomolecule motion in environments mimicking experimental conditions are our main tools to evaluate the behavior of the inference. Here, we focus our simulations on the most relevant situations met experimentally. We also expand partially to conditions that may be found in other experiments.

C.1 Notations and comments for hopping simulations

In the rest of the supportinginformation MAP stands for Maximum *A Posteriori* and Pdf for Probability density function. Except where indicated otherwise, the double-well potentials used for the simulations in 1D are

$$V(x)=\frac{\Delta E}{X_+^4}x^4-\frac{2\Delta E}{X_+^2}x^2$$

with ΔE the hopping energy and 0 and X_+ and $-X_+$ the positions of the two well minima. The double-well potentials used for the simulations in 2D are

$$V(x,y)=\frac{\Delta E}{X_+^4}x^4-\frac{2\Delta E}{X_+^2}x^2+ky^2$$

with ΔE the hopping energy, X_+ and X_+ the positions of the two well minima, and k the spring constant of the harmonic confinement in the y-axis direction. The y-axis confinement will be referred to as lateral confinement. For escaping events from a unique well in both 1D and 2D the confinement potentials are harmonic and symmetrical. Outside the region of confinement the potential value is set to 0 k_BT.

We define the confinement factor *u* as in Ref. [3]. For one confinement well $u=D\Delta t/L^2$, with D the average diffusivity, Δt the time between frames and L the characteristic diameter of the well. For two confining wells $u=D\Delta t/L^2$ with D the maximal value of the average diffusivity in the two wells and L the characteristic diameter of the smaller confining well.

Positioning noise is introduced in the simulations with Gaussian noise. This noise is used to model all the sources of noise found experimentally, *i.e.* Poisson noise due to the signal and the fluorescent background, camera read-out noise, and noise due to the positioning algorithm and the imperfect modeling of the Point Spread Function.Noise in the potential fields is introduced as a sum of cosine and sinus functions (section C.4).

In simulations with varying diffusivity, the diffusivity takes a common value for $|x|>X_+$ and another one for $|x|<X_+$. In simulations with a unique hopping event, trajectories are generated in the same manner as for all the other simulations; only the ones with a unique hopping event are analyzed.

In all the cases where we claim that the bias can be corrected, we mean that by performing simulations in conditions matching experimental ones, a curve or multidimensional surface of the bias evolution with the parameters involved can be extracted and the bias can be corrected by extracting all the inferred parameters from the trajectoryas in Ref. [3].

Finally, each probability density function (Pdf) in the main paper and in the supporting information was obtained from at least 2000 (from 2000 to 10000) numerically generated trajectories.

C.2 Choice of the estimator

In order to extract hopping energies, we chose the maximum of the *a posteriori* probability (MAP) as the estimator. In previous work (Ref. [4]), we chose (or focused on) the average value estimator (AVE) of the *posteriori* distribution. In the present case, the MAP outperforms other estimators (Figure S7).

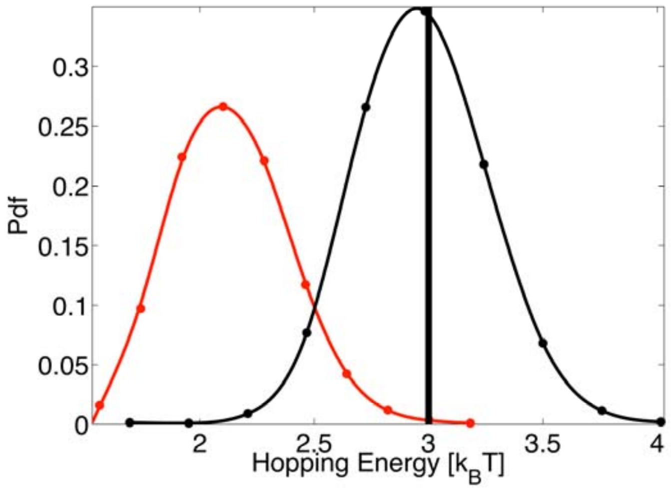

Figure S7: Pdf of the inferred hopping energy for the MAP estimator (Black) and for the average value of the *posteriori* probability distribution estimator (AVE, Red) for hopping between two confining wells in 2D. 2000 point trajectories, 3 k_BT theoretical hopping energy

(shown by the thick vertical black line), 200 nm between the two wells, 0.025 $\mu m^2.s^{-1}$ diffusivity and 25 ms acquisition time.

The MAP estimator yields a distribution centered on the theoretical energy used in the simulations (Fig. S7). Biases in the AVE are mostly due to asymmetries in the *posteriori* distribution favoring higher diffusivities in the mesh squares and concomitantly shifting the hopping energy towards lower values. This phenomenon of drifting of inferred hopping energies towards lower values due to higher diffusivities is often encountered in these kinds of inference [3].

Because we are not using the AVE, we cannot access the error in the hopping energy by sampling the *posteriori* distribution. Furthermore, we cannot evaluate the Fisher Information [4] due to the large set of possible realizations. Therefore, the error in the hopping energy estimation is derived from the statistics of MAP results for numerical trajectories matching experimental conditions.

C.3 Characteristics of the MAP statistics

We here investigate the tail of the hopping energy MAP statistics (30000 trajectories generated and analyzed with inference).

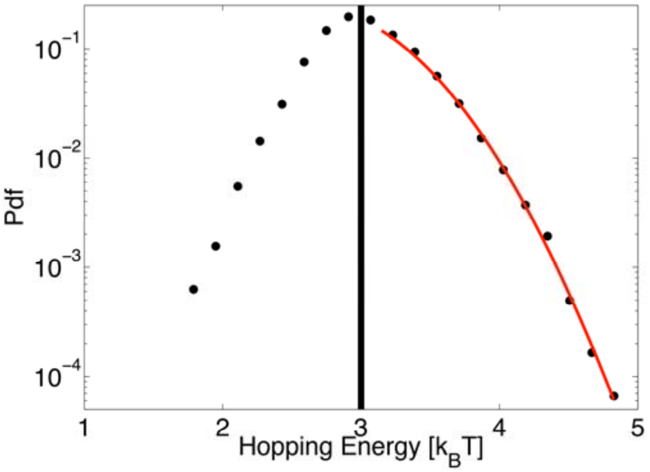

Figure S8: Pdf of the MAP for the hopping energy between two confining wells in 2D in log scale. The red curve is the asymptotic Gaussian decay. 2000 points trajectories, 3 k_BT theoretical hopping energy (shown by the thick vertical black line), 400 nm between the two wells, 0.025 $\mu m^2.s^{-1}$ diffusivity and 25 ms acquisition time.

The statistics exhibit a fast decreasing behavior both for low and high hopping energy values (Fig. S8). For high hopping energy values, the statistics evolves as $\exp(-(E_h/0.58)^2)$. Thus, the significant values of the inference are well centered around the maximum of the MAP statistics and there is no long-tail behavior.

C.4 Noise in the potential fields

The biomolecule may experience the combined effect of general potential and local interactions of inferior magnitude leading to a potential with noise structures. The noise

structures can also be considered as the consequence of multiple small potential barriers. We simulated trajectories in a double-well potential both in 1D and in 2D with additional sinusoidal terms to model the substructures, with ε the standard deviation of the potential noise and constant diffusivity.

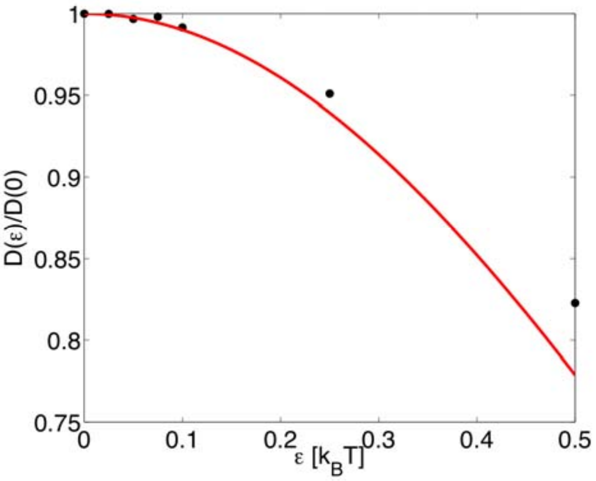

Figure S9: Evolution of the inferred diffusion coefficient (normalized to the no-noise limit) with the standard deviation of the potential noise for 1D double-well trajectories. The black dots are the average values of the MAP statistics and the red line is the Zwanzig model [5] that models the effect of potential noise on the diffusivity. 2000-point trajectories, 3 k_BT theoretical hopping energy, 400 nm between the two wells, 0.025 $\mu m^2.s^{-1}$ diffusivity and 25 ms acquisition time.

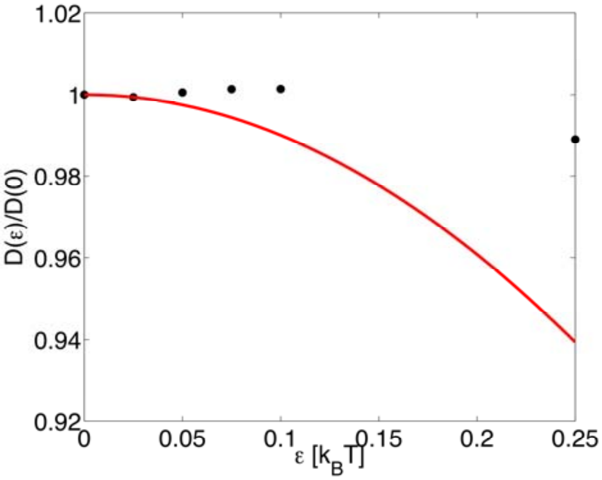

Figure S10: Evolution of the inferred diffusion coefficient (normalized to the no-noise limit) with the standard deviation of the potential noise for 2D double-well trajectories. The black dots are the average values of the MAP statistics and the red line is the 1D Zwanzig model[5]. 2000-point trajectories, 3 k_BT theoretical hopping energy, harmonic confinement along the y-axis with spring constant 200 $k_BT.\mu m^{-2}$, 300 nm between the two wells, 0.035 $\mu m^2.s^{-1}$ diffusivity and 25 ms acquisition time.

The inference does not capture the substructures of the potential. However, the inferred diffusivities diminished with rising amplitude of the potential noise following the equation: $D(\varepsilon)/D(0)=\exp(-\varepsilon^2/(k_BT)^2)$. This result was predictable and has already been demonstrated by

Zwanzig[5] who showed that noise in the potential is reflected in the diffusivity. The data in Figs. S9 and S10 show good agreement with the Zwanzig formula for 1D inference. In 2D, a different evolution is shown. To our knowledge, there are no general models describing the evolution of diffusivity with noise in 2D potentials. Note that evolutions tend to be extremely sensitive to the nature of the potential noise.

C.5 Evolution of the inference with a unique hopping event allowed

Experimental recordings often catch a unique hopping event. We study the MAP Pdf of the hopping energy with aunique hopping event limitation in 2D.

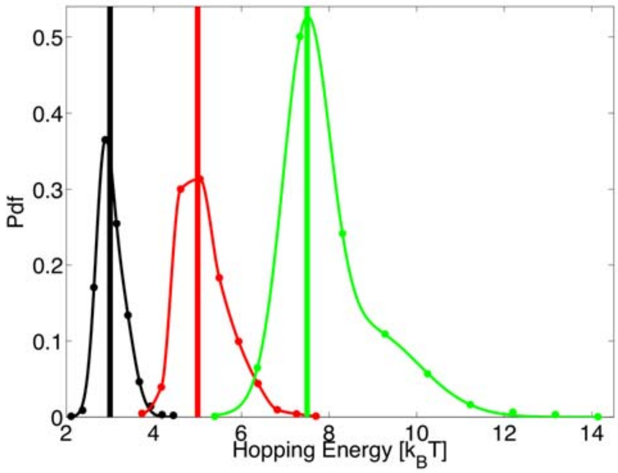

Figure S11: Evolution of the MAP Pdf with hopping energy for trajectories with a unique hopping event. 3 k_BT hopping energy in black, 5 k_BT hopping energy in red and 7.5 k_BT hopping energy in green. 2000-point trajectories, 300 nm between the two wells, 0.025 $\mu m^2.s^{-1}$ diffusivity and 25 ms acquisition time.

For various conditions, when the estimator is unbiased with multiple hopping events, it remains unbiased for a unique hopping event. The main consequence of the unique hopping event limitation is the broadening the Pdf.

C.6 Effect of lateral confinement in 2D motion on the inference

Here, we study the effect of lateral confinement on the MAP Pdf of the hopping energy.

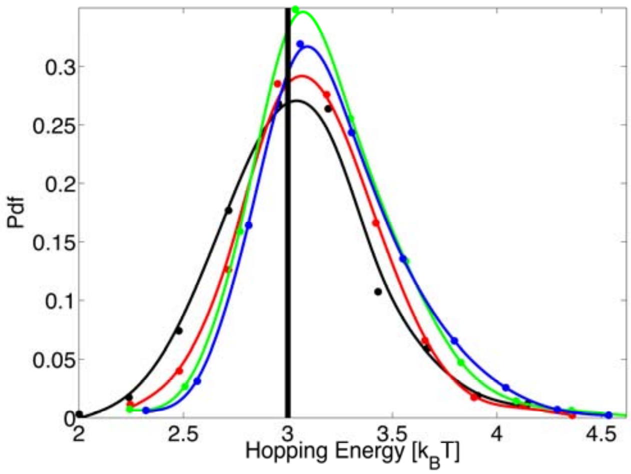

Figure S12: Evolution of the MAP Pdf with lateral confinement spring constant for k=50 $k_BT.\mu m^{-2}$(black), k=100 $k_BT.\mu m^{-2}$(red), k=200 $k_BT.\mu m^{-2}$(green), k=250 $k_BT.\mu m^{-2}$(blue). 2000-point trajectories, 3 k_BT theoretical hopping energy (shown by the thick vertical black line), 0.035 $\mu m^2.s^{-1}$ diffusivity, 400 nm between the two wells, and 25 ms acquisition time.

There are no major effects on the statistics for large sets of lateral confinements. This effect confirms that, for various experimental conditions, 1D and 2D trajectories lead to the same results and that, therefore, 1D simulations may catch most of the information necessary to characterize the inference behavior.

C.7 Effects of diffusivity on the inference of hopping energies between two wells

a) Uniform diffusivity in 1Dtrajectories

Certain diffusivity values and experimental conditions leading to a high confinement factor, u, may generate bias on the inference in the case of simple confinement [3]. These effects can be compensated in most cases. Here, we show the effect of the rise of the global diffusivity on the inference for 1D trajectories (Fig. S13). Similar effects of lesser magnitude are observed for 2D trajectories.

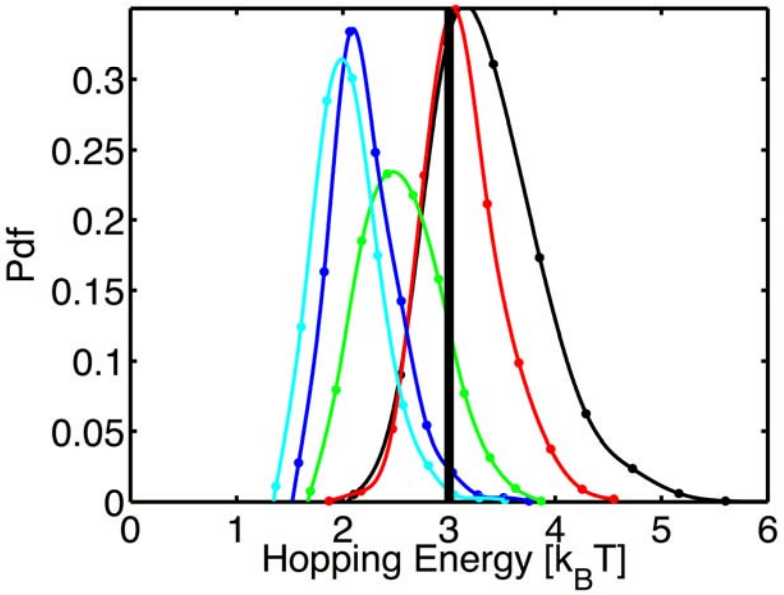

Figure S13: Evolution of the MAP Pdf of the hopping energy with the diffusivity for a 1D double-well potential. D=0.01 μm^2.s^{-1} in black, D=0.02 μm^2.s^{-1} in red, D=0.04 μm^2.s^{-1} in green, D=0.075 μm^2.s^{-1} in blue, D=0.1 μm^2.s^{-1} in cyan. 2000-point trajectories, 3 k_BT theoretical hopping energy (vertical Black line), 400 nm between the two wells, and 25 ms acquisition time.

Interestingly, despite the rise of diffusivity and consequently confinement factor u inside each well,we still obtain good inferences. As the diffusivity rises, the inferred hopping energies decrease. The bias evolution can be fitted by a second-order polynomial and thus compensated (Fig. S14). The drift of hopping energy with diffusivity always shows the same behavior. The drift curve depends on the value of the hopping energy, the level of confinement inside each well, and the distance between the two wells. Whereas confinement was a major issue when dealing with motion inside a unique well,in the double-well case, information can be retrieved for higher confinement factors u than for a unique well confinement.

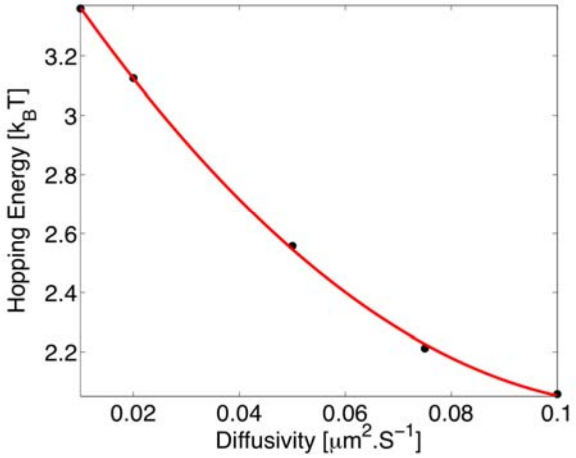

Figure S14: Evolution of the average MAP values with diffusivity. The black dots are the results of the inferences and the red line is aparabolic fit.

b) Varying diffusivities in 1D trajectories

Here, the diffusivitiesare identical in the two wells but are higher in the central zone between the two wells ($|x| < X_+$). As the diffusivity in the central hopping zone rises, the inferred value of the hopping energy shifts towards lower values. The shift is deterministic, always lowering the inferred value of the hopping energy with rising central diffusivities. Therefore, this bias can be corrected.

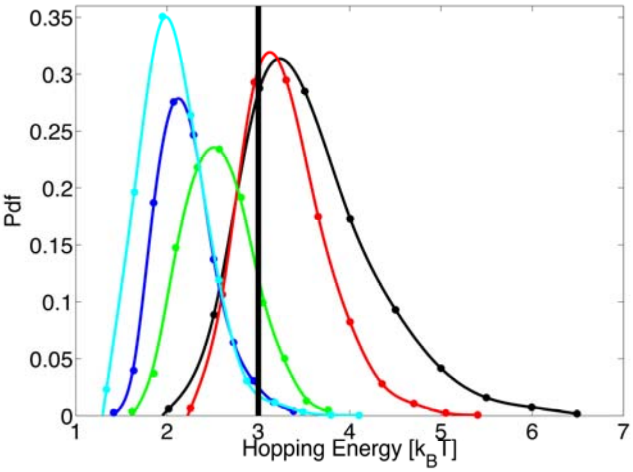

Figure S15: Evolution of the MAP Pdf with varying central diffusivity for a 1D double-well potential. D=0.00625 $\mu m^2.s^{-1}$ in black, D=0.0125 $\mu m^2.s^{-1}$ in red, D=0.05 $\mu m^2.s^{-1}$ in green, D=0.075 $\mu m^2.s^{-1}$ in blue, D=0.125 $\mu m^2.s^{-1}$ in cyan. 2000-point trajectories, 3 k_BT theoretical hopping energy (shown by the thick vertical black line), diffusivities in the wells D=0.025 $\mu m^2.s^{-1}$, 400 nm between the two wells, and 25 ms acquisition time.

c) Varying diffusivities in 2D trajectories

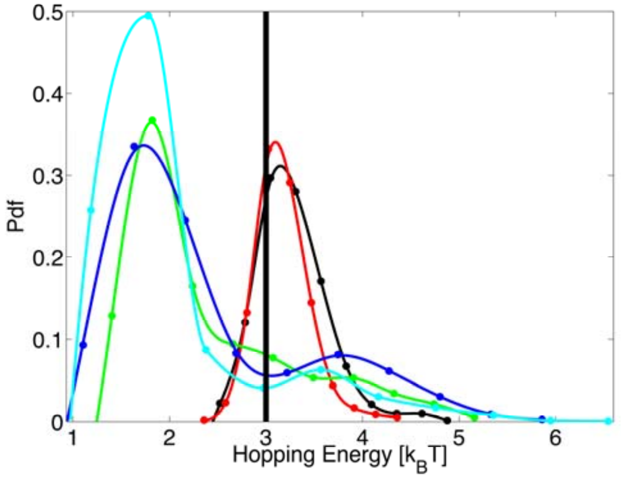

Figure S16: Evolution of the MAP Pdf with varying central diffusivity for a 2D double-well potential. D=0.00625 μm^2.s^{-1} in black, D=0.0125 μm^2.s^{-1} in red, D=0.05 μm^2.s^{-1} in green, D=0.075 μm^2.s^{-1} in blue, D=0.125 μm^2.s^{-1} in cyan. 2000-point trajectories, 3 k_BT theoretical hopping energy (shown by the thick vertical black line), diffusivities in the wells D=0.035 μm^2.s^{-1}, 400 nm between the two wells, and 25 ms acquisition time.

Here also, the diffusivities are identical in the two wells but are higher in the central zone between the two wells ($|x| < X_+$).The change of central diffusivity may have a strong effect on the inference. We here show a unique set of simulation results. Extended simulations, however, have shown that these biases are sensitive to the diffusivity inside the wells, to the distance between the wells, to the hopping energy and to the nature of the lateral confinement. Therefore, when dealing with varying diffusivities, large amounts of simulations must be performed to catch the effect of the different parametersof the environment on the inference and the resulting biases. However, these biases can be compensated.

C.8 Escaping from a Unique Well to Free Motion

Experimentally, none of the toxin receptors exhibited pure escaping behavior from unique wells. Yet, this is an important case that appears in many other biological systems: the biomolecule escapes from a confinement area and then diffuses freely (or under a much more shallow potential). In these cases, the same inference scheme, as the one used for the double well trajectories, is used on the trajectories; however, the two main issues are the definition of the domain size and the definition of the hopping energy (see section A). In the following simulations, the size of the confinement domain and the shape its limits are supposed to be known.

a) 1D trajectories

The hopping energy is defined as the higher of the two energies at the limit of the domain. We have shown, in the main text, the behavior of the inference with the hopping energy assuming that the diffusivity is identical inside and outside the well. Here, we show for a unique hopping energy the evolution of the MAP statistics with a differentexternal diffusivity.

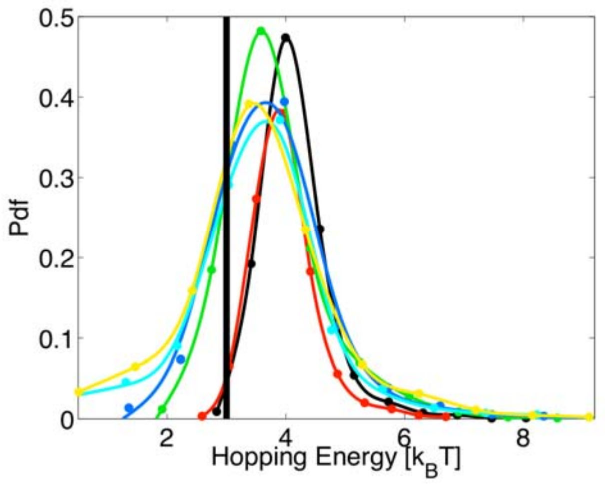

Figure S17: Evolution of the MAP Pdf with the external diffusivity. D=0.0088 μm^2.s^{-1} in black, D=0.0175 μm^2.s^{-1} in red, D=0.07 μm^2.s^{-1} in green, D=0.105 μm^2.s^{-1} in blue, D=0.175 μm^2.s^{-1} in cyan and D=0.35 μm^2.s^{-1} in yellow. 2000-point trajectories with at least 1000 points inside the well, 3 k_BT theoretical hopping energy (shown by the thick vertical black line), diffusivity inside the well D=0.035 μm^2.s^{-1}, 100 nm radius of the well, and 25ms acquisition time.

In the unique well escaping inference, the external diffusivity does not modify strongly the bias but mainly broadens the Pdf. Here again, bias and evolution are sensitive to the diffusion inside the well, the size of the well and the shape of the potential.

b) 2D trajectories

Here, we show for a unique hopping energy the evolution of the MAP statistics with changing external diffusivity.The evolution is similar to the one for 1D trajectories. The major effect of the rise of external diffusivity is the broadening of the MAP Pdf.

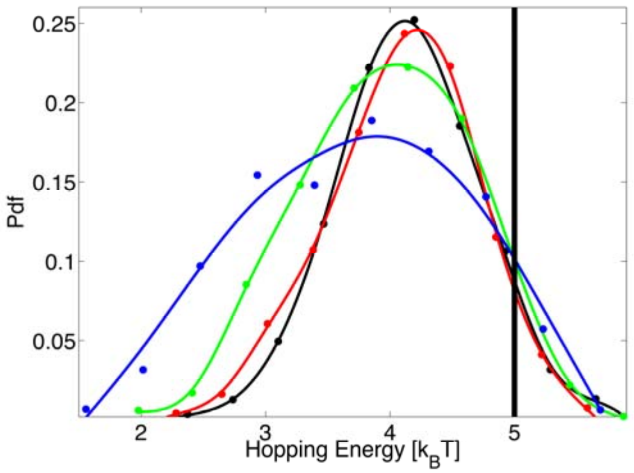

Figure S18: Evolution of the MAP Pdf with external diffusivity. D=0.0125 μm^2.s^{-1} in black, D=0.05 μm^2.s^{-1} in red, D=0.125 μm^2.s^{-1} in green, and D=0.25 μm^2.s^{-1} in blue. 1000-point trajectories with at least 500 points in the well, 5 k_BT theoretical hopping energy (shown by the thick vertical black line), diffusivity inside the well D=0.025 μm^2.s^{-1}, 100 nm radius of the well, and 25 ms acquisition time.

C.9 1D vs. 2D simulations

In many simulation conditions, we noticed that inferences performed on 1D simulated trajectories lead to the same results as inferences performed on 2D trajectories. In these cases, as extensive simulations are needed to evaluate noise and bias on the inferred parameters, 1D simulations which are much faster to compute may be used to capture the essential features of the inference performance.

D. Experimental hopping

D.1Identification of the hopping events

In order to eliminate the possibility of fast local motion of the membrane that could be assimilated to hopping, hopping events were only considered as such if locally, in the recorded image, the other receptors did not exhibit any sudden motion or hopping-like events.

D.2Correctingharmonic bias

When performing the inference in the harmonic approximation of the potential, some bias appears during optimization. We mentioned this effect in Ref. [3], showed its evolution with the confinement factor u and how to correct it. Here, we display the inferred values of diffusivity and spring constant as a function of the input values of diffusivity and spring constant when the inference is performed on numerically generated trajectories.

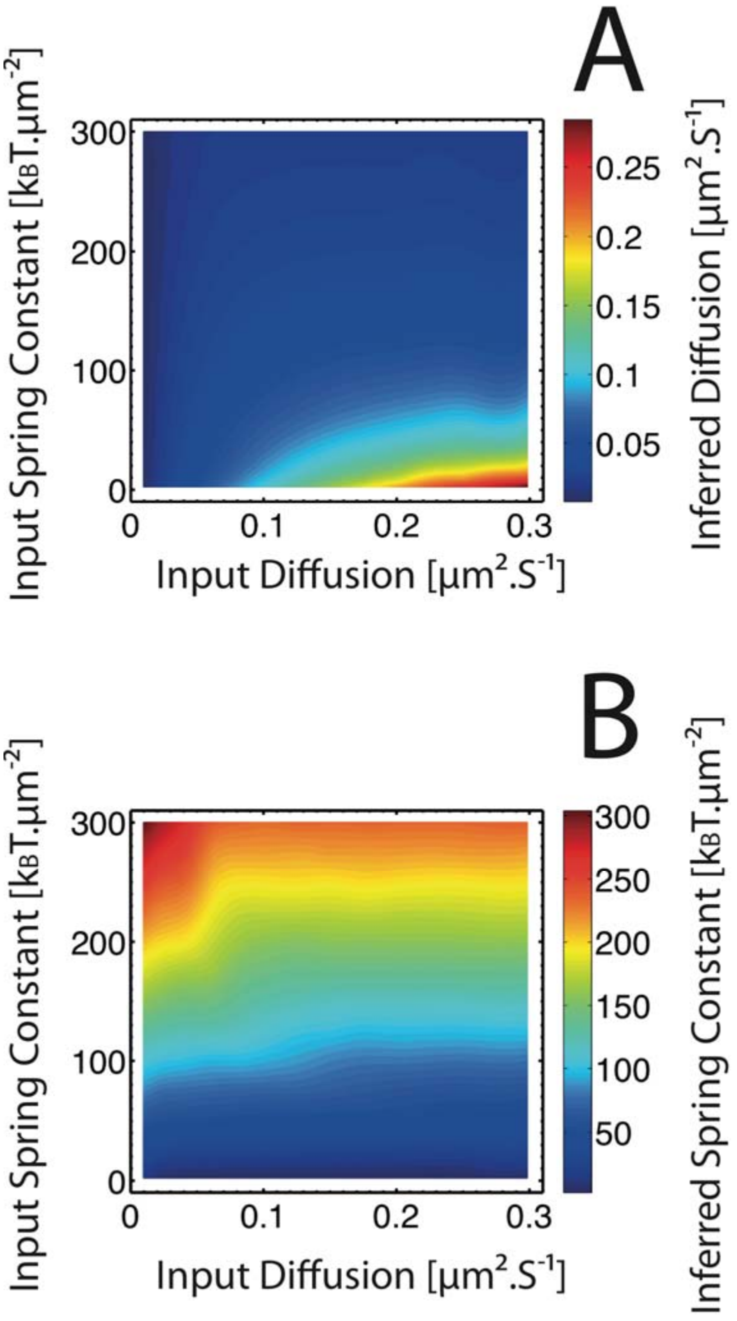

Figure S19: Evolution of the inferred value of the diffusivity (A) and spring constant (B) with the input diffusivity and spring constant used in the numerical simulations of the trajectories.

These two plots allow correction of all the spring constant and diffusivity values inferred from experimental trajectories.

D.3Examples of two-wellpotentials

a) Low hopping energies

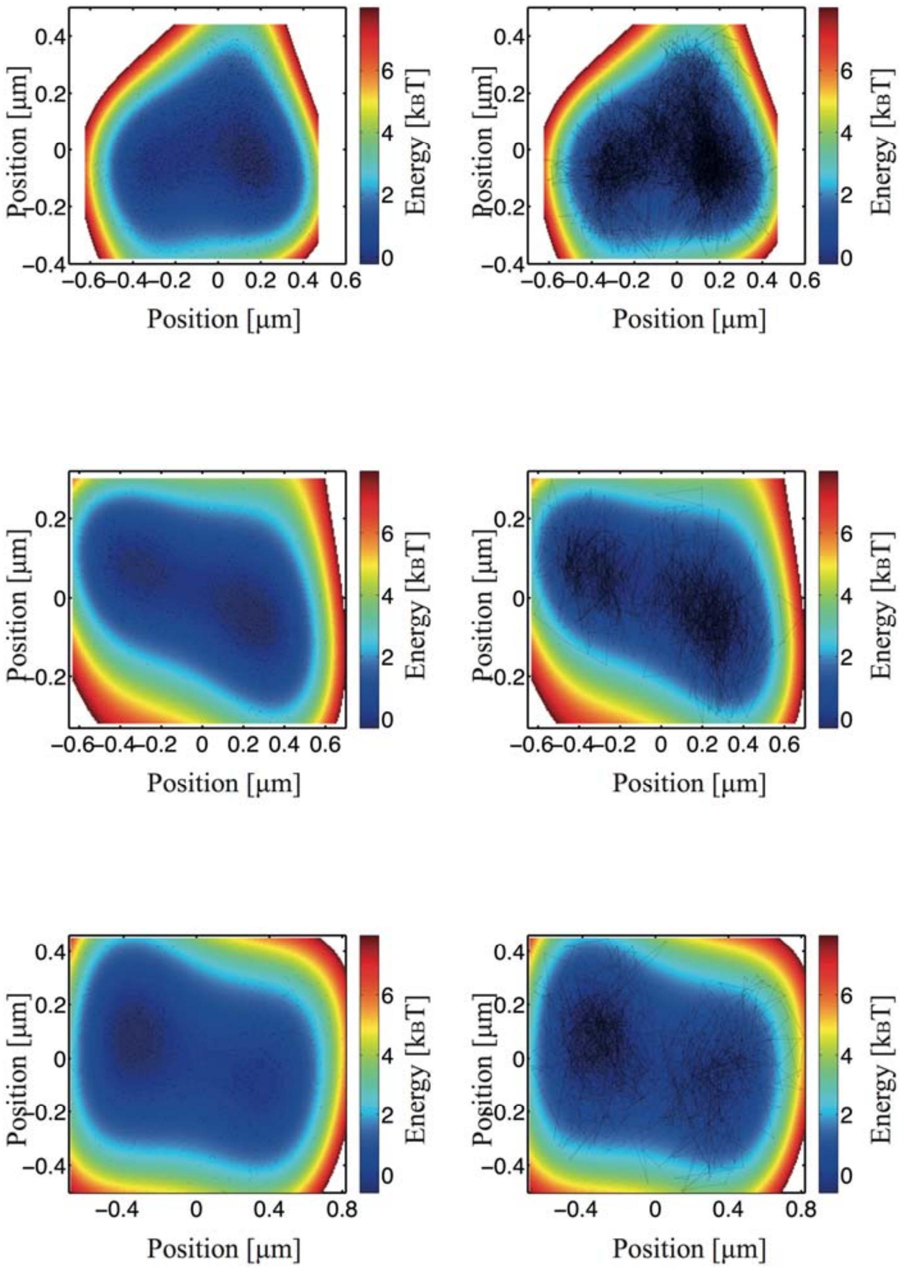

Figure S20: Three examples of two-well potentialswith low hopping energy between the two wells. On the left, only the trajectory points are superimposed on the image; on the right, the trajectory points are linked to materialize the trajectory. The inferred hopping energies are from top to bottom: 0.47, 0.26, and 0.43 k_BT.

b) High hopping energies

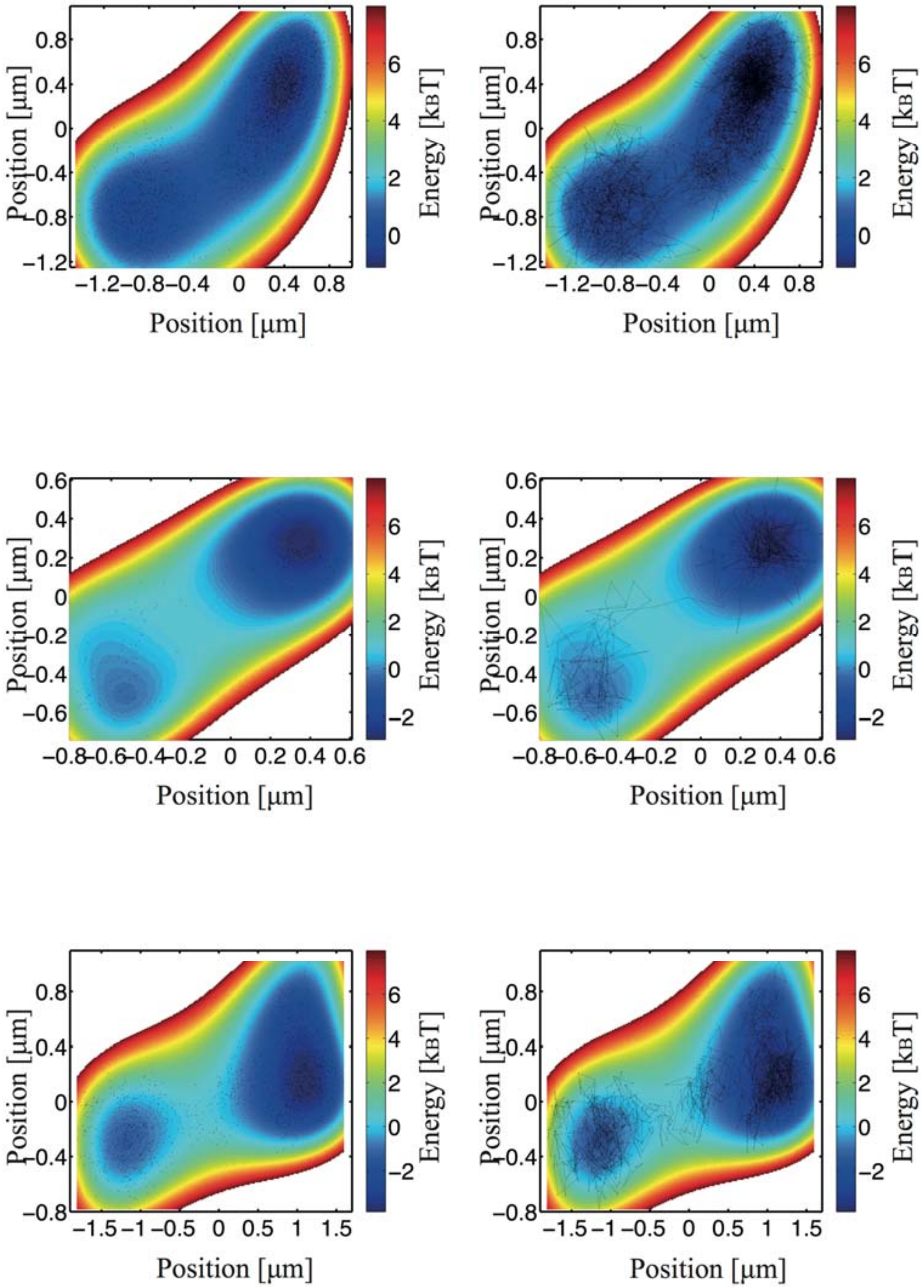

Figure S21: Three examples of two-well potentialswith high hopping energy between the two wells. On the left, only the trajectory points are superimposed on the image; on the right, the trajectory points are linked to materialize the trajectory. The inferred hopping energies are from top to bottom: 1.7, 1.2, and 2.3 $k_B T$.

c) Figure 4 with materialization of the trajectory

We show the diffusivity and potential fields of Figure S22 with connected trajectory points to materialize the trajectory.

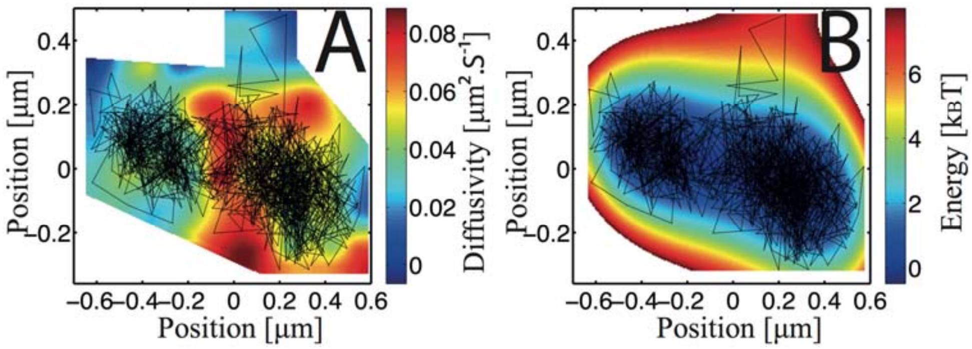

Figure S22: Diffusivity and Potential Map with the full visible trajectory of the receptor corresponding to Figure 4.A) Diffusivity map of the membrane area where the receptor moves. The diffusivity field was generated by abi-harmonic interpolation of the inferred diffusivity field on the mesh. B) Inferred interaction energy map felt by the receptor. Black lines connect the successive positions of the biomolecule.

d) Intermediate structures

In some cases, the inferred potential exhibited a main well and a lateral extension without a second local minimum. We designate this type of potential a confinement well with intermediate structures. An example of these structures is shown in Fig. S23.

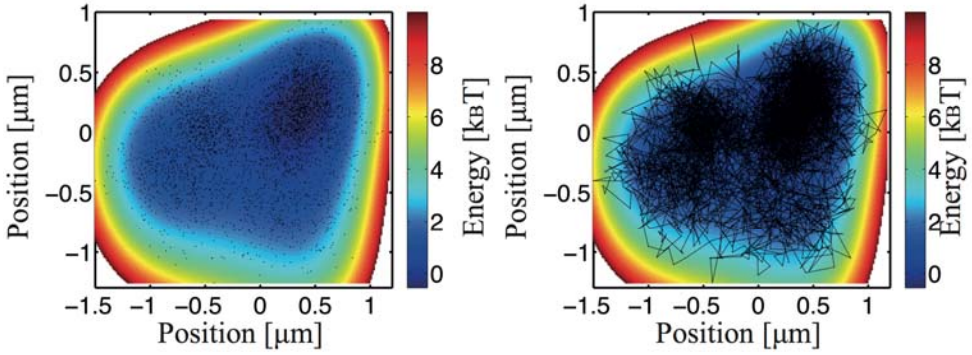

Figure S23: Interaction potential acting on the ε-toxin receptor inferred from a 4262-point trajectory. On the left, only the trajectory points are superimposed on the image; on the right, the trajectory points are linked to materialize the trajectory.

15 trajectories exhibited this type of structure. Interestingly, these potentials do not show a second minimum, yet there are two well-defined areas of the potential. These potential structures suggest that the larger rafts may be formed by coalescence of smaller rafts that undergo lipid reorganization. The two-well potentials with small hopping energies that we observe may be linked to early stages in the coalescence process, where the lipid organization

of the rafts is still similar to that of isolated ones. Experimental trajectories on even longer time scales are necessary to fully confirm that these are indeed coalescence processes. The main difficulty is the related to the local motion of the cell membrane that prevents efficient, reliable use of the inference.

References

[1] Türkcan S, Masson J-B, Casanova D, Mialon G, Gacoin T, et al. (2012) Observing the confinement potential of bacterial pore-forming toxin receptors inside rafts with non-blinking Eu^{3+} -doped oxide nanoparticles. Biophys J 102: 2299-2308

[2] Ehrensperger MV, Hanus C, Vannier C, Triller A, Dahan M (2007) Multiple Association States between Glycine Receptors and Gephyrin Identified by SPT Analysis. Biophys J 92: 3706-3718

[3] Türkcan S, Alexandrou A, Masson J-B (2012) A Bayesian inference scheme to extract diffusivity and potential fields from confined single-molecule trajectories. Biophys J 102: 2288-2298

[4] Voisinne G, Alexandrou A, Masson J-B (2010) Quantifying Biomolecule Diffusivity Using an Optimal Bayesian Method. Bio Phys J 98:596-605

[5] Zwanzig R (1988) Diffusion in a rough potential. Proc Natl Acad Sci USA 85:2029-2030
